# Exogenous proline enhances susceptibility of NSCLC to cisplatin *via* metabolic reprogramming and PLK1-mediated cell cycle arrest

**DOI:** 10.3389/fphar.2022.942261

**Published:** 2022-07-14

**Authors:** Bingjie Han, Yuanyuan Sun, Xiaofen Zhang, Ping Yue, Meiling Tian, Dan Yan, Fanxiang Yin, Bo Qin, Yi Zhao

**Affiliations:** ^1^ Department of Translational Medical Center, the First Affiliated Hospital of Zhengzhou University, Zhengzhou, China; ^2^ Department of Obstetrics and Gynecology, the First Affiliated Hospital of Zhengzhou University, Zhengzhou, China

**Keywords:** cisplatin resistance, metabolic reprogramming, NSCLC, proline, plk1

## Abstract

The occurrence of cisplatin resistance is still the main factor limiting the therapeutic effect of non-small cell lung cancer (NSCLC). It is urgent to elucidate the resistance mechanism and develop novel treatment strategies. Targeted metabolomics was first performed to detect amino acids’ content in cisplatin-resistant cancer cells considering the relationship between tumour metabolic rearrangement and chemotherapy resistance and chemotherapy resistance. We discovered that levels of most amino acids were significantly downregulated, whereas exogenous supplementation of proline could enhance the sensitivity of NSCLC cells to cisplatin, evidenced by inhibited cell viability and tumour growth *in vitro* and xenograft models. In addition, the combined treatment of proline and cisplatin suppressed ATP production through disruption of the TCA cycle and oxidative phosphorylation. Furthermore, transcriptomic analysis identified the cell cycle as the top enriched pathway in co-therapy cells, accompanied by significant down-regulation of PLK1, a serine/threonine-protein kinase. Mechanistic studies revealed that PLK1 inhibitor (BI2536) and CDDP have synergistic inhibitory effects on NSCLC cells, and cells transfected with lentivirus expressing shPLK1 showed significantly increased toxicity to cisplatin. Inhibition of PLK1 inactivated AMPK, a primary regulator of cellular energy homeostasis, ultimately leading to cell cycle arrest via FOXO3A-FOXM1 axis mediated transcriptional inhibition in cisplatin-resistant cells. In conclusion, our study demonstrates that exogenous proline exerts an adjuvant therapeutic effect on cisplatin resistance, and PLK1 may be considered an attractive target for the clinical treatment of cisplatin resistance in NSCLC.

## 1 Introduction

Lung cancer is the most common and leading cause of cancer-related mortality worldwide (Bray et al., 2018). Among them, non-small cell lung cancer (NSCLC) is the primary histological subtype and accounts for about 85% of lung cancer cases (Meza et al., 2015). Recently, targeted therapy has improved the therapeutic effect and outcomes in patients with NSCLC, whereas platinum-based combination chemotherapy is still used as the standard first-line treatment (Planchard et al., 2019; de Mello et al., 2020). However, adaptive resistance occurs when the tumour cells survive after early treatment(Bivona and Doebele, 2016). Thus, the chemotherapy effects are limited by the development of cisplatin resistance. Therefore, it is urgent to understand the underlying mechanism involved in mediating chemoresistance and propose appropriate treatment strategies for restoring the cisplatin sensitivity of NSCLC.

It has been confirmed that cisplatin resistance is closely associated with the alterations of multiple cellular processes, including DNA repair, antioxidant system, cell cycle, drug transport system and other molecular signal pathways (Gentile et al., 2016; Sarin et al., 2017; Kou et al., 2020). In addition, the most prominent mode of action of cisplatin is considered to induce interstrand and intrastrand cross-linking of tumour cells, thereby inhibiting cell replication and transcription and ultimately leading to apoptosis or necrosis caused by DNA damage response (Gonzalez et al., 2001; Galluzzi et al., 2012; Zhang et al., 2014). The changes of most oncogenes and tumour suppressors in these signaling pathways appear to affect cellular metabolic pathways, allowing cancer cells to convert ATP to amino acids, nucleotides, fatty acids and other intermediates conducive to tumour progression (Boroughs and DeBerardinis, 2015; Wishart, 2015).

As accumulating research focuses on the effect of tumour metabolism on chemoresistance, the relationship between amino acid metabolism and cisplatin resistance is further elucidated. In previous studies, a wide range of metabolic changes is detected in cisplatin resistance cells compared with parental cells of NSCLC. Alteration of the glutathione metabolic pathway closely relates to cisplatin sensitivity (Obrist et al., 2018; Shi et al., 2019). Moreover, deprivation of lysine enhanced cisplatin cytotoxicity, and their combination inhibited the growth of ectopic hepatomas by increasing cellular apoptosis and DNA damage (Wahwah et al., 2020). The other studies also prove that cancer cells with or without chemoresistance depend on specific amino acids, such as serine, glycine, aspartate, proline and kynurenine (Jain et al., 2012; Lukey et al., 2017; Sullivan et al., 2018; Liu et al., 2020; Muthusamy et al., 2020; Nguyen et al., 2020). However, it remains unclear which relevant amino acids and molecular targets participate in cisplatin susceptibility of NSCLC.

To gather insights, we performed integrated omics, including targeted metabolomics and transcriptomics, to systematically analyze the mechanism of amino acids regulated therapeutic efficacy of NSCLC. Metabolic requirements for amino acids of various diseases and significant changes in metabolites related to amino acid circulation pathways under stress can be analyzed through targeted metabolomics (Garcia−Bermudez et al., 2018). Combined with transcriptomics, molecular changes, and cellular events induced by amino acid changes can be further identified. Since amino acid metabolism is linked to the cell cycle, targeting the serine/threonine-protein kinase PLK1 as a cell cycle regulator has been proven a viable therapeutic strategy against cancer chemoresistance (Jain et al., 2012; Shao et al., 2015; Wu et al., 2019). PLK1 is highly overexpressed in various cancer cells, and inhibition of PLK1 leads to mitotic arrest and cell apoptosis (Xu et al., 2021). However, the cellular effects of PLK1 in cisplatin-resistant NSCLC cells have not been studied.

In the current study, we observed that proline functions as a promoter of cisplatin sensitivity for NSCLC both *in vitro* and *in vivo*. Exogenous proline alters the chemoresistance of cancer cells through disruption of multiple metabolic pathways at the transcriptome level, especially for the energy production and cell cycle. Moreover, inhibition of PLK1 enhanced the cytotoxic effect of cis-diamminedichloroplatinum (II) (CDDP) on chemoresistant cells. These findings raised the possibility that PLK1 inhibition induced by supplementation of proline in synergy with cisplatin *in vivo* might be a promising strategy for treating cisplatin resistance in NSCLC.

## 2 Materials and methods

### 2.1 Tumour cell culture

A549 (CVCL_0023) and A549/CDDP cells were cultured in RPMI 1640 medium (Gibco, C11875500BT, United States ) supplemented with 10% FBS (Gibco, 10100147, AUS), 100 units/mL penicillin, and 100 units/mL streptomycin (Gibco, 15140122) at 37°C in 5% CO_2_. H460 (CVCL_0459) and A2780 (CVCL_0134) cells were obtained from ATCC and cultured under the same condition as A549. All the corresponding cisplatin resistance cells were established as described previously (Peter et al., 1992). 2 μM cisplatin (Sigma-Aldrich, P4394, CAS Number: 15,663-27-1) was added to the medium to maintain the CDDP resistance. HEK-293T cells were grown in Dulbecco’s modified Eagle’s medium supplemented with 10% FBS in the presence of antibiotics in a humidified incubator at 37°C.

### 2.2 Sample preparation of targeted metabolomics of amino acids

A549 and A549/CDDP cells were seeded in 10 cm dishes and grown to approximately 90% confluency (*n* = 7). Cells were then harvested and washed twice with PBS, and metabolites were extracted using cold 40/40/20 (v/v/v) methanol/acetonitrile/water. In addition, prepare one more sample from each group for cell counting. For amino acids analysis, evaporated samples were reconstituted in water containing 0.1% formic acid (FA), followed by acetonitrile containing 0.1% FA. Then, the samples were separated by Agilent 1,290 Infinity UPLC system in Shanghai Applied Protein Technology Co., Ltd. (Shanghai, China). Mobile phase A was 25 mM ammonium formate containing 0.08% FA. Mobile phase B was acetonitrile having 0.1% FA. The flow rate was 250 μl/min, and the injection volume was 1 μl. The gradient conditions were as follows: MPB linearly changed from 90 to 70% at 12 min, decreased to 50% at 18 min, then decreased to 40% at 25 min, followed by linearly changed from 40 to 90% at 30 min, held at 90% MPB for 7 min. The total run time was 37 min. Data were acquired using QTRAP 5500 (AB SCIEX) mass spectrometry in positive ionization mode, and MRM mode was used to detect the ion pairs. The raw files were imported to Multiquant software to extract the peak area and retention time. The amino acids and derivatives were identified using the corresponding isotopolog.

### 2.3 Transcriptomic analysis

Total RNA of A549, A549/CDDP control groups, 10 μM CDDP-treated groups, 20 mM proline-treated groups, and the combination-treated groups were isolated using TRIzol reagent (Invitrogen). RNA concentration was measured using a Qubit RNA assay kit in Qubit 2.0 fluorometer (Life Technologies, CA, United States ). The purity and quality of RNA were monitored by 1% agarose gel electrophoresis.

Briefly, the initial preprocessing of the raw intensity data was firstly processed through in-house Perl scripts to remove reads with low quality, and then the trimmed reads were aligned against the reference genome of humans using TopHat (v2.0.4) and using the gene annotation available at Ensembl (v77). Differential expression analysis between two groups was performed using the DESeq R package (v1.18.0). The *p*-values were adjusted using Benjamini and Hochberg’s approach for controlling the false discovery rate. Genes with adjusted *p*-value < 0.05 and |FoldChange| ≥ 1.5 observed by DESeq were assigned as differentially expressed. Finally, the samples were analyzed by principal component analysis (PCA) and cluster analysis for differential genes. Subsequent enrichment analyses, including Gene Ontology (GO) and Kyoto Encyclopedia of Genes and Genomes (KEGG) pathway enrichment, were performed by DAVID bioinformatics resources (v6.8).

### 2.4 Combination index

The cell proliferation after BI2536 (Selleck, S1109, CAS Number: 755038-02-9) and CDDP combined-treatment were detected by MTT assay according to the method described in Supporting Information. Then the effects of the drug combination were calculated using the combination index (CI) through CalcuSyn software(Chou, 2006). Antagonism is indicated when CI > 1, CI = 1 indicates an additive effect, and CI < 1 indicates synergy.

### 2.5 Lentiviral transfection

A549 and A549/CDDP cells were transfected with the PLK1 pLKO5-puro-shPLK1 expression plasmid using Lipofectamine 3,000 (Invitrogen, L3000015) according to the manufacturer’s recommendation. The sequences used were listed in Supplementary Table S2. Cells were infected by adding the lentiviral particles to the culture medium with 10 μg/ml polybrene. For generating stable knockdown cells, cells were treated with 8 μg/ml puromycin for 72 h after the transduction and maintained for more than 14 days.

### 2.6 Mice study

To assess the effect of proline on the cisplatin response *in vivo*, 30 female BALBc/nu mice (4–6 weeks old; 16–18 g weight) were randomly divided into six groups (A549 group, *n* = 5; A549 with cisplatin injection group, *n* = 5; A549/CDDP group, *n* = 5; A549/CDDP with proline administration group, *n* = 5; A549/CDDP with cisplatin injection group, *n* = 5; A549/CDDP with combination injection group, *n* = 5). The mice were subcutaneously injected with 2 × 10^6^ cells (A549, A549/CDDP) near the axillary fossa. Cisplatin (3 mg/kg) and proline (20 g/kg) were dissolved in 0.9% sterile normal saline and injected into the tumour twice a week when the tumour volume had reached approximately 1,000 mm^3^. Mice were humanely sacrificed by euthanasia after treatment. Due to the slow growth of tumours derived from A549/CDDP cells, the xenograft tumours were harvested after 10 weeks. Then, the tumour volumes were measured according to the following formula: tumour volume = length × (width)^2^/2.

### 2.7 Histological analysis

Tumour tissues collected from other groups of mice were fixed in 10% neutral buffered formalin and embedded in paraffin. Tumour tissues were then cut into 4 μm thick sections, and the sections were dewaxed. The sections were then stained with hematoxylin and eosin (H&E) (Servicebio, Wuhan, China). Stained sections were used to observe the structure and cellular morphology of tumour tissues under a digital scanner (Panoramic 250) and were analyzed by CaseViewer software (version 2.3).

### 2.8 Immunohistochemistry staining

IHC staining of PLK1 was performed according to the manufacturer’s instructions in the previous study (Yang et al., 2018). In brief, samples resected from mice were fixed overnight and then stored in 70% ethanol before processing. The primary antibody was added to the tissue sections blocked in serum for incubating overnight at 4°C. Sections were further incubated with biotin-conjugated secondary antibody for 1 h, then treated with SABC, 3,3-diaminobenzidine (DAB) and hematoxylin staining. After staining, the sections were dehydrated and mounted in a permount mounting medium (Fisher Scientific). Finally, the images were scanned with a digital scanner (Panoramic 250) and viewed by CaseViewer software (version 2.3).

### 2.9 Ethics statement

All animal maintenance and procedures were conducted following the National Institutes of Health Guide for the Care and Use of Laboratory Animals and were approved by the Animal Care and Ethics Committee of First Affiliated Hospital of Zhengzhou University (2021-KY-0856).

### 2.10 Statistical and data analysis

Three biological replicates were performed for each experiment. The results are shown as means ± standard error of the mean (SEM) and were statistically analyzed using GraphPad Prism software version 8.0 (GraphPad Software, Inc, La Jolla, CA, United States ). One-way ANOVA and Two-tailed Student t-test were used to compare the values of the test and control samples, and *p*-value of *p < 0.05, **p < 0.01, ***p < 0.001 were considered statistically significant. The details of other biological assays are described in Supplementary Materials and Methods.

## 3 Results

### 3.1 Targeted metabolomics reveals alteration of amino acids in cisplatin-resistant NSCLC cells

**FIGURE 1:**
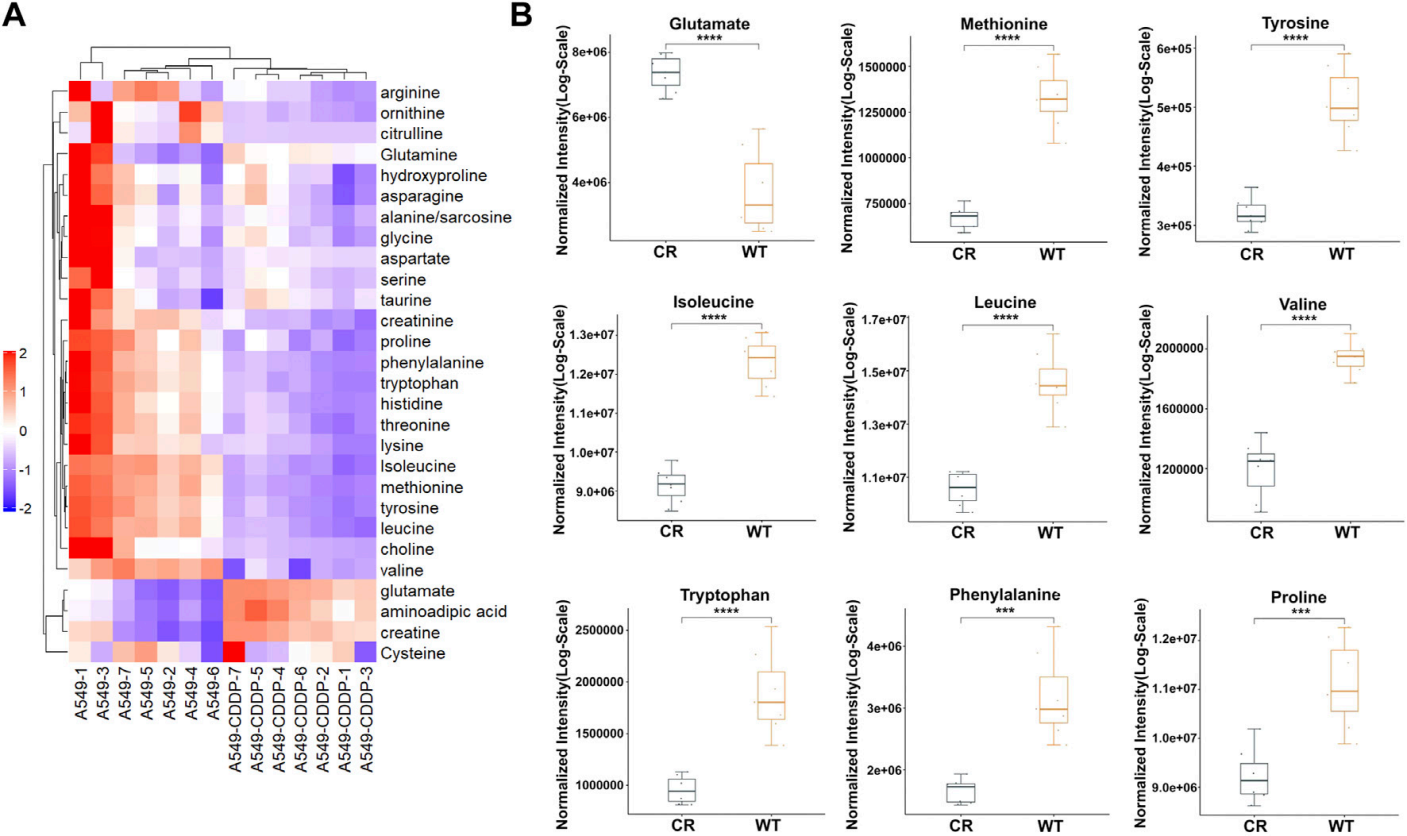
The amino acid targeted metabolic profiling reveals significantly altered metabolites in A549 cells with cisplatin resistance. **(A)** Heatmap of differential amino acids quantified in A549 and A549/CDDP cells. **(B)** Boxplots of the relative concentration distribution of representative differential amino acids, including glutamate, methionine, tyrosine, isoleucine, leucine, valine, tryptophan, phenylalanine, and proline. *p < 0.05, **p < 0.01, ***p < 0.001, ****p < 0.0001.

To track the types of amino acids that affect cisplatin resistance in NSCLC, targeted amino acid metabolomics based on UPLC-MS were performed in cisplatin-sensitive A549 cells and cisplatin-resistant A549/CDDP cells. According to the RSD distribution map of QC samples, the experimental detection process and results were stable and dependable (Supplementary Figure S1A). The results showed that 18 amino acids with a *p*-value < 0.05 were significantly different in abundance among the total detected 28 amino acids (Figure 1A). Among these, the concentration of three amino acids was significantly upregulated in A549/CDDP cells, including glutamate, aminoadipic acid, and creatine. Another 15 amino acids include isoleucine, methionine, valine, leucine, tyrosine, tryptophan, phenylalanine, threonine, histidine, proline, creatinine, lysine, ornithine, choline and arginine were identified with decreased abundance (Figure 1B, Supplementary Figure S1B).

Since the abundance of most amino acids in cisplatin resistance cells was significantly down-regulated, we hypothesized that the amino acid metabolism was disrupted in A549/CDDP cells. KEGG pathway analysis was examined through MetaboAnalyst (version 5.0) to assess the affected amino acid metabolic pathways. Six pathways associated with amino acids were identified to be enriched, including tyrosine metabolism, histidine metabolism, cysteine and methionine metabolism, tryptophan metabolism, phenylalanine metabolism, and phenylalanine, tyrosine, and tryptophan biosynthesis (Supplementary Figure S1C).

### 3.2 Exogenous proline re-sensitizes NSCLC cells with cisplatin resistance

**FIGURE 2:**
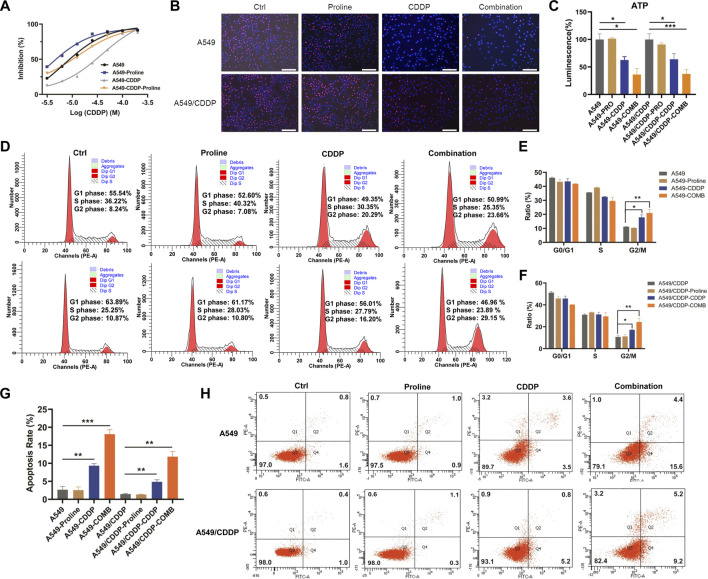
Proline supplementation significantly enhanced the sensitivity of cancer cells to cisplatin. **(A)** The viability of control or proline-treated A549 and A549/CDDP cells exposed to different concentrations of cisplatin for 72 h was analyzed by MTT assay. **(B)** EdU assay for the effect of 20 mM proline alone or combined with 10 μM CDDP on the proliferation of A549 and A549/CDDP cells after 48 h *in vitro*. **(C)** The abundance of ATP in A549 and A549/CDDP cells treated with 20 mM proline, 10 μM CDDP, and their combination for 72 h. **(D)** The cell cycle distribution of A549 (up) and A549/CDDP (down) cells after 20 mM proline, 5 μM CDDP, and the combination treatment for 48 h was detected by flow cytometry. **(E,F)** Quantification analysis of cell cycle arrest in **(D)**. **(G,H)** Analysis of apoptosis in A549 and A549/CDDP cells exposed to 20 mM proline, 5 μM CDDP, and their combination for 72 h by flow cytometry. EdU: 5-ethynyl-2′-deoxyuridine. *p < 0.05, **p < 0.01, ***p < 0.001.

Cancer cells with increasing nutrient consumption display an unusual metabolic state to satisfy the demands of rapid cell growth and proliferation, whereas the cancer cell shows dependency on specific amino acids(Otto Warburg et al., 1927; Cairns et al., 2011; Cocetta et al., 2020). Cells exhibited different metabolic responses with the exogenous addition of significantly differential amino acids. The inhibition effect of cisplatin in the presence of valine, leucine, and phenylalanine on A549 and A549/CDDP cells had no significant difference from that of cisplatin alone (Supplementary Figure S2A); regardless of cisplatin concentration, the addition of 6.4 mM tryptophan, 6 mM tyrosine, and 8 mM glutamate significantly inhibited cell growth (Supplementary Figure S2B). The sensitivity of parental and cisplatin-resistant A549 cells to cisplatin was enhanced only by 20 mM proline supplementation for 72 h, and was also validated on H460, H460/CDDP, A2780 and A2780/CDDP cells (Figure 2A, Supplementary Figure S3). The IC_50_ values of cisplatin in A549 and A549/CDDP cells were changed from 6.7 to 38.8 μM–3.1 and 12.8 μM in the absence and presence of proline, respectively. The combination of proline with cisplatin resulted in a further decrease in the proportion of EdU-positive cells compared with cells exposed to proline or cisplatin alone, implying inhibition of cell proliferation (Figure 2B, Supplementary Figure S4). In addition, significant decreases in ATP levels were detected after co-treatment with CDDP and proline, suggesting that the cellular energy metabolism was disturbed by proline supplementation (Figure 2C).

To further investigate the mechanism of action of proline, we performed cell cycle and apoptosis assay by flow cytometry. The results revealed that the combination of proline and CDDP led to a further significant increase in the G2 phase compared with the CDDP-treated group, whereas the addition of proline alone did not affect the cell cycle of both A549 and A549/CDDP cells (Figure 2D). Notably, the accumulation of the G2 phase was accompanied by a decrease in the S phase in A549 cells (Figure 2E), whereas in A549/CDDP cells, G2/M arrest occurred with a reduction in the G1 phase (Figure 2F). Furthermore, exposure to CDDP combined with proline induced dramatic cellular apoptosis in sensitive and resistant cell lines (Figures 2G,H).

### 3.3 Proline combined with CDDP induces global changes of the transcriptome in NSCLC

**FIGURE 3:**
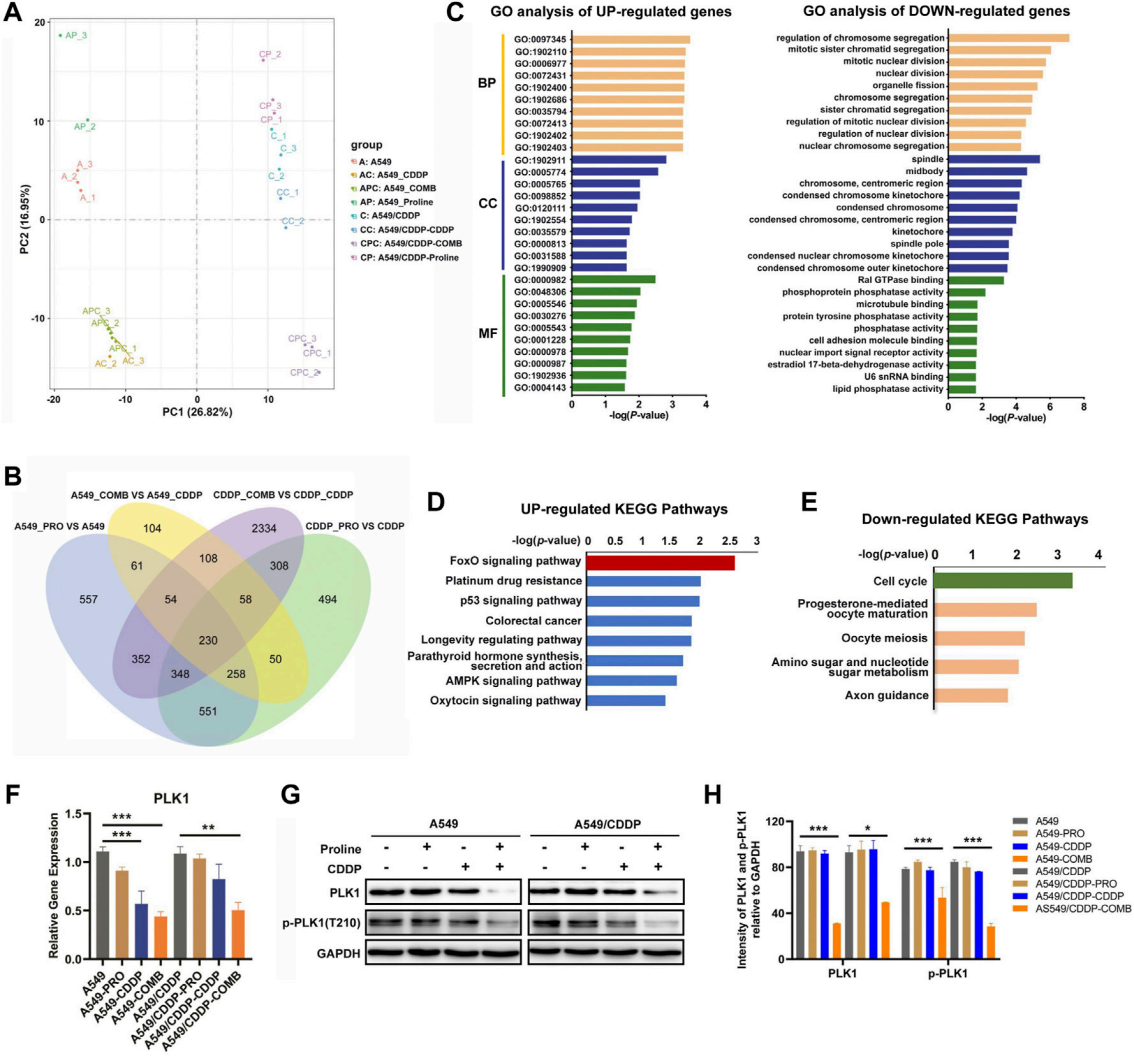
Transcriptomic profiling reveals the alteration of gene transcription levels in A549 and A549/CDDP cells upon 20 mM proline, 10 μM CDDP, and their combination treatment. **(A)** PCA analysis of transcriptomic profiles obtained from A549 and A549/CDDP cells exposed to different treatments. Each dot represents a biological repeat. **(B)** Venn plot shows the overlap of significantly differential genes of four groups (group 1/2/3/4) in A549 and A549/CDDP cells. **(C)** GO analysis was performed on 108 significantly differential genes corresponding to **(B)**. **(D,E)** KEGG enrichment pathway analysis of upregulated and downregulated mRNAs in **(B)**. **(F)** Relative transcription levels of PLK1 in A549 and A549/CDDP cells were detected by qRT-PCR. **(G)** Effects of proline, CDDP, and their combination on protein levels of p-PLK1 and PLK1. **(H)** The bands in **(G)** were quantified and presented as the mean ± SEM of three independent experiments. Statistical significance was determined by a two-tailed, paired Student’s t-test. *p < 0.05, **p < 0.01, ***p < 0.001.

We then evaluated the transcriptomic profiles of A549 and A549/CDDP cells exposed to 20 mM proline, 10 μM CDDP, and their combination for 24 h to explore the underlying mechanisms by which proline enhanced cisplatin sensitivity. The principal components analysis (PCA) results showed a clear separation between eight groups (Figure 3A). One hundred eight significantly differential genes induced by proline were identified in four comparisons, including proline combined with CDDP-treated A549 versus CDDP-treated A549 (group 3) and proline combined with CDDP-treated A549/CDDP versus CDDP-treated A549/CDDP (group 4), whereas excluding proline-treated A549 versus A549 (group 1) and proline-treated A549/CDDP versus A549/CDDP (group 2) (Figure 3B). After eliminating 17 genes that showed differential expression between group 3 and group 4, 50 upregulated and 41 down-regulated genes were used for Gene Ontology (GO) and Kyoto Encyclopedia of Genes and Genomes (KEGG) pathway analysis. The representative upregulated molecular functions of GO terms were significantly enriched in DNA damage response and signal transduction, whereas the down-regulated genes enriched GO terms were regulation of chromosome segregation (Figure 3C). The KEGG pathway enrichment analysis revealed that increased DEGs participated in various metabolic pathways, such as FoxO signaling pathway, platinum drug resistance, p53 signaling pathway and AMPK signaling pathway (Figure 3D). Significantly down-regulated genes were enriched in several metabolic pathways, with cell cycle enrichment being the most significant (Figure 3E). Apart from that, the differentially upregulated and down-regulated genes identified in group 3 and group 4 were related to a broader range of metabolic changes through GO and KEGG pathway analysis (Supplementary Figure S5, Supplementary Figure S6).

Since the cell cycle was the predominant signaling pathway affected by down-regulated genes, it may contribute to the susceptibility of cisplatin in NSCLC. We firstly detected the transcript levels of the down-regulated genes involved in the cell cycle by qRT-PCR, including serine/threonine-protein kinase (*PLK1*), G2/mitotic-specific cyclin-B1 (*CCNB1*), M-phase inducer phosphatase 2 (*CDC25B*) and securin (*PTTG1*). The results showed that only the expression level of *PLK1* was significantly decreased, consistent with transcriptomic profiles (Figure 3F, Supplementary Figure S7). Meanwhile, the western blotting assay confirmed that the protein expression levels of PLK1 and p-PLK1 decreased after co-therapy of proline and CDDP (Figures 3G,H). We further investigated the PLK1 expression in other cell models, including cisplatin-sensitive and cisplatin-resistant H460 lung cancer cells and A2780 ovarian cancer cells. The expression patterns of PLK1 in A549 were consistent with that in H460 and A2780 cells exposed to the combination of proline and CDDP (Supplementary Figures S8). The above data suggested that PLK1 played a critical role in mediating cisplatin susceptibility.

### 3.4 Exogenous proline mediates sensitivity of NSCLC to cisplatin in a xenograft model

**FIGURE 4:**
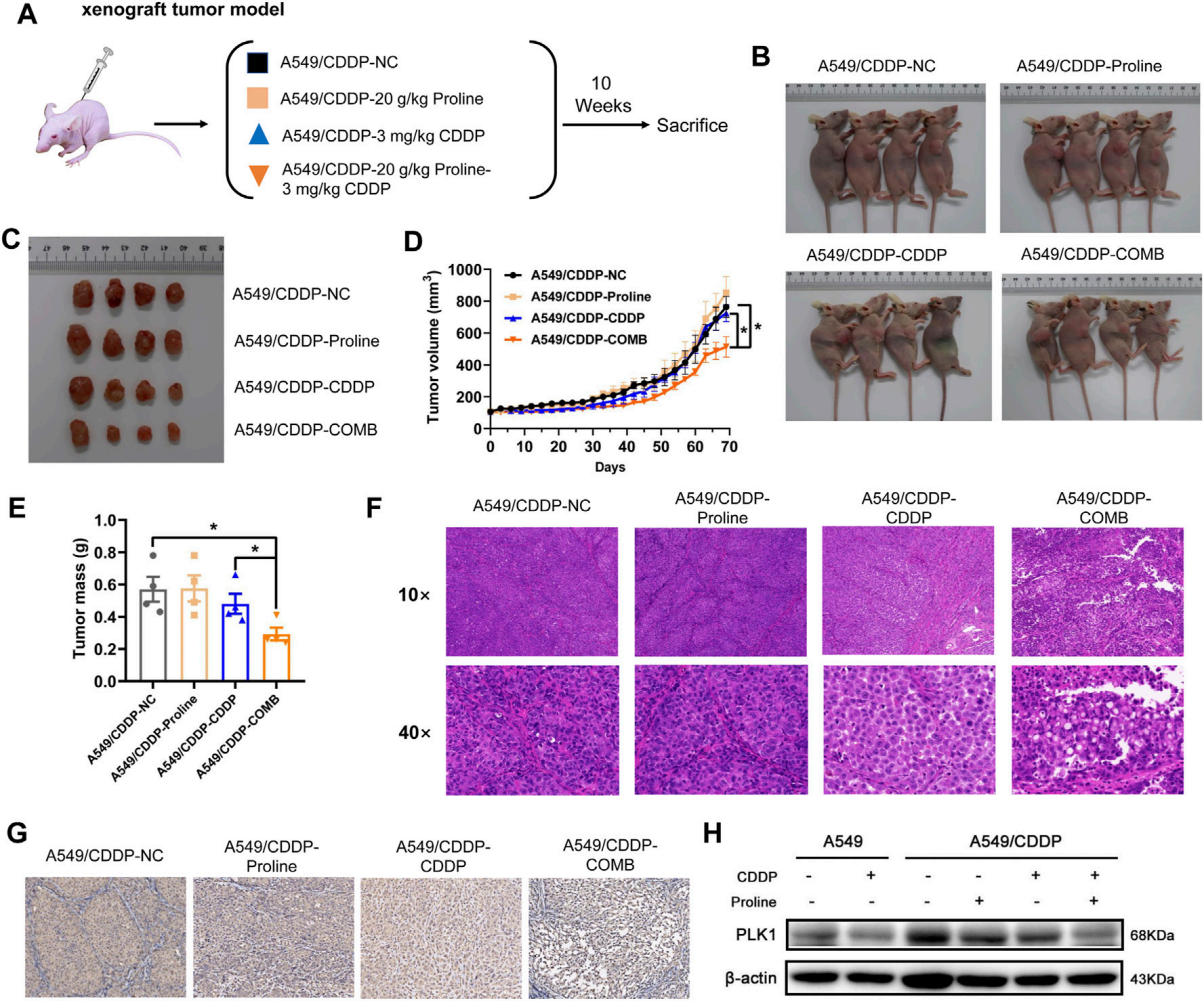
Combination of proline and cisplatin results in NSCLC growth inhibition *in vivo*. **(A)** The xenograft tumour model investigates the effects of 20 g/kg proline, 3 mg/kg cisplatin and their combined therapy on cisplatin-resistant A549 cells. **(B)** Representative imaging of mice 10 weeks after injection of A549/CDDP cells upon different treatments. **(C)** Combined treatment of proline and CDDP effectively inhibited A549/CDDP cell’s subcutaneous tumour growth in nude mice. **(D)** The tumour volume was monitored every 3 days to generate the tumour growth curves. **(E)** The tumours were extracted and weighed. **(F)** H&E-stained tumour sections. **(G)** Sections of tumours were stained with PLK1 antibody by immunohistochemical analysis. **(H)** Detection of protein expression levels of PLK1 in various groups by western blotting. The data are the means ± SEMs of three independent experiments. *p < 0.05.

We then determined whether the combination of proline and cisplatin could attenuate the chemoresistance of NSCLC *in vivo*. A549 and A549/CDDP cells were injected subcutaneously into the underarms of nude mice to generate xenograft models. Mouse xenograft models were then treated with CDDP (3 mg/kg) every 3 days, proline (20 g/kg) every day, and combination therapy every 3 days (Figure 4A). The PBS-treated group was used as a control. Mice were monitored each day, and tumour growth was measured every 3 days. As shown in Figures 4B–E, the combination therapy of proline and CDDP resulted in significantly enhanced inhibition of tumour growth, tumour weights, and volumes compared with monotherapy. The results showed that the tumour growth rate in the A549/CDDP group was lower than that in the parental group, and the inhibitory effect of cisplatin on the tumour derived from the A549 cells was significantly more substantial than that of the cisplatin-resistant group (Supplementary Figures S9A–D). In addition, the combination of cisplatin and proline did not inhibit tumours derived from A549/CDDP cells to the same extent as cisplatin did for A549 cell implantations.

Furthermore, histological analyses of tumour sections by HE staining indicated that the combination of proline and cisplatin triggered extensive tumour necrosis compared with the control tissue sections, and free cisplatin also induced somewhat tumour necrosis compared with the control group (Figure 4F). In A549 cells exposed to cisplatin, similar to the A549/CDDP cells upon combination treatment of proline and cisplatin, the standard cell structures were not visible, cells were loosely arranged in adjacent necrotic areas, and nuclei were ruptured and deeply stained (Supplementary Figure S9E). IHC results showed that the combination of exogenous proline and cisplatin significantly reduced the staining of PLK1 in A549/CDDP tumour xenograft compared with their individual effects (Figure 4G, Supplementary Figure S9F, Supplementary Figure S9G). Consistently, the addition of proline led to decreased protein expression levels of PLK1 in xenografts, as detected by western blotting (Figure 4H, Supplementary Figure S9H). These data support the idea that the addition of exogenous proline might improve the sensitivity of NSCLC cells to cisplatin by inhibiting the expression of PLK1.

### 3.5 PLK1 inhibition decreases CDDP resistance in NSCLC by inducing cell cycle arrest

**FIGURE 5:**
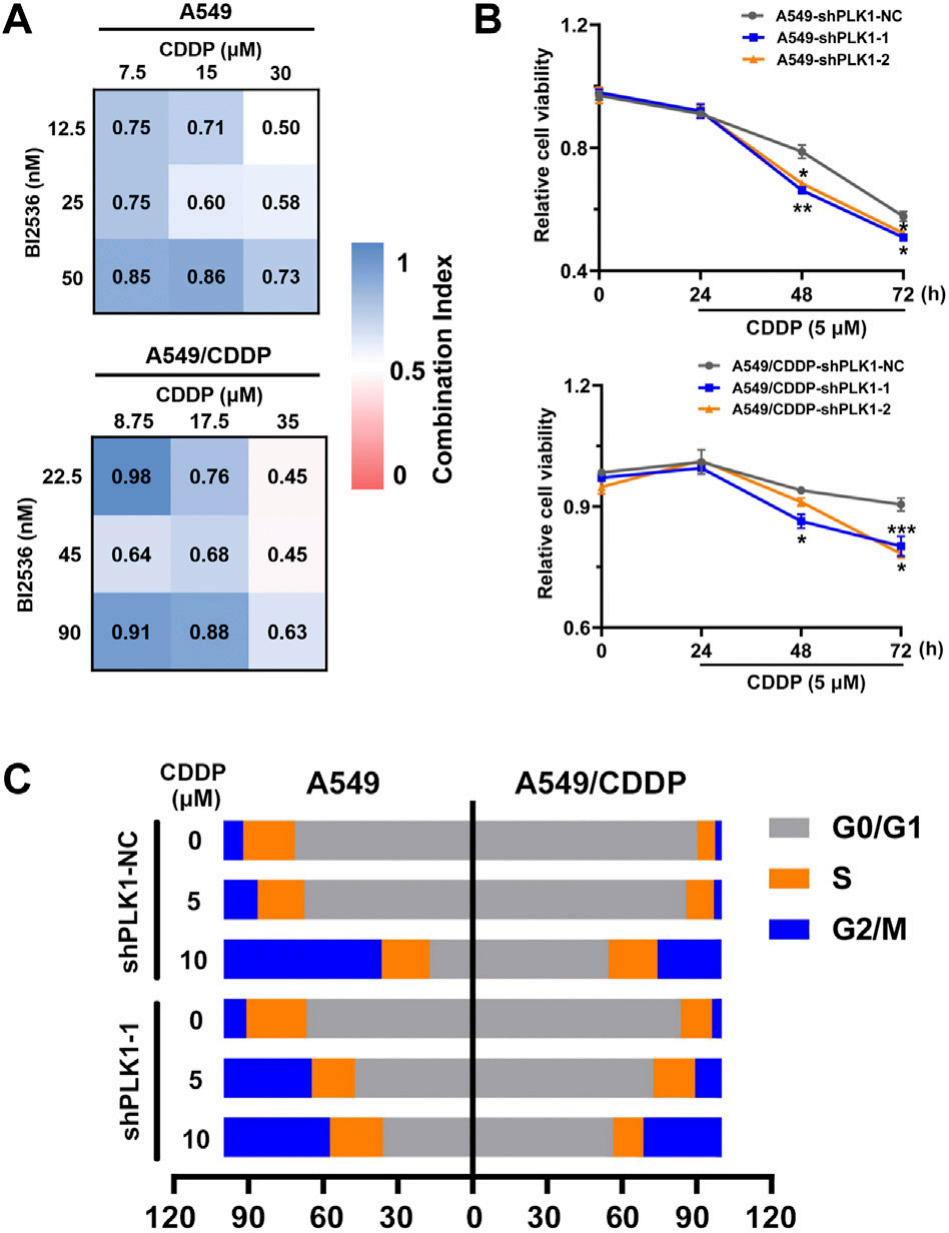
The knockdown of PLK1 enhances the efficacy of CDDP by inducing cell cycle arrest. **(A)** CI heatmaps of A549 (up) and A549/CDDP (down) cells were treated with BI2536 and CDDP at the indicated doses. **(B)** MTT assays were performed to assess cell viability of A549 and A549/CDDP cells stably transfected with PLKO.5 and PLK1 shRNA with or without 5 μM cisplatin treatment for 24, 48, and 72 h. **(C)** The cell cycle distribution in NC shRNA- and PLK1 shRNA-transfected cells treated with or without 5 μM cisplatin was detected by flow cytometry.

To further investigate the molecular mechanism of PLK1 regulation, we first detected the synergistic effects of the PLK1 inhibitor BI2536 and CDDP on A549 and A549/CDDP cells. The synergic effect was exhibited by CI values, which were determined through the Chou-Talalay method using CompuSyn software(Chou, 2006), with the CI values <1 indicating a synergistic effect between two drugs. They had synergistic effects when A549 cells were treated with BI2536 at concentrations ranging from 12.5 to 50 nM and CDDP at concentrations ranging from 7.5 to 30 μM, and when A549/CDDP cells were treated with BI2536 at concentrations ranging from 22.5 to 90 nM and CDDP at concentrations ranging from 8.75 to 35 μM (Figure 5A). A549 and A549/CDDP cells were then transfected lentivirus expressing with empty vector and PLK1-shRNA to clarify the functional role of PLK1. qRT-PCR and western blotting analyses showed that the expression of PLK1 was efficiently blocked in PLK1-shRNA lentivirus infected cells (Supplementary Figure S10A, Supplementary Figure S10B). Cell viability of PLK1-knockdown cells was further suppressed compared with the cells transfected with an empty vector under 5 μM cisplatin treatment for 48 or 72 h (Figure 5B). This was based on the fact that the growth curves of the PLK1 depleted group had no significant difference from those of the control group (Supplementary Figure S10C, Supplementary Figure S10D). PLK1 inhibition, especially in cells transfected with plasmid shPLK1-2, suppressed the cell colony formation ability, including number and size, implying that downregulation of PLK1 inhibited cell proliferation (Supplementary Figures S10E).

Considering the relevance of PLK1 to the cell cycle, we further performed FACS analysis to determine the changes in cell cycle distribution in A549 and A549/CDDP cells transfected with PLK1-shRNA lentivirus upon exposure to cisplatin. The data showed that the percentage of G2/M phase in A549 cells infected with Scr-shRNA increased from 7.91 to 13.65% upon 5 μM cisplatin treatment, whereas the proportion in the cells infected with PLK1-shRNA significantly rose from 9.13 to 35.39%, accompanied by a decrease in G0/G1 phase (Supplementary Figure S11). In addition, the percentage of G2/M phase in A549/CDDP cells transfected with lentivirus expressing PLK1-shRNA increased from 3.89 to 10.60% under 5 μM cisplatin treatment, whereas no significant change in the Scr-shRNA infected groups was present (Figure 5C). These data suggested that PLK1 silence significantly enhanced the sensitivity of NSCLC cells to cisplatin by mediating cell cycle arrest at the G2/M phase.

### 3.6 The FOXO3A-FOXM1 axis participates in PLK1-regulated cell cycle progression

**FIGURE 6:**
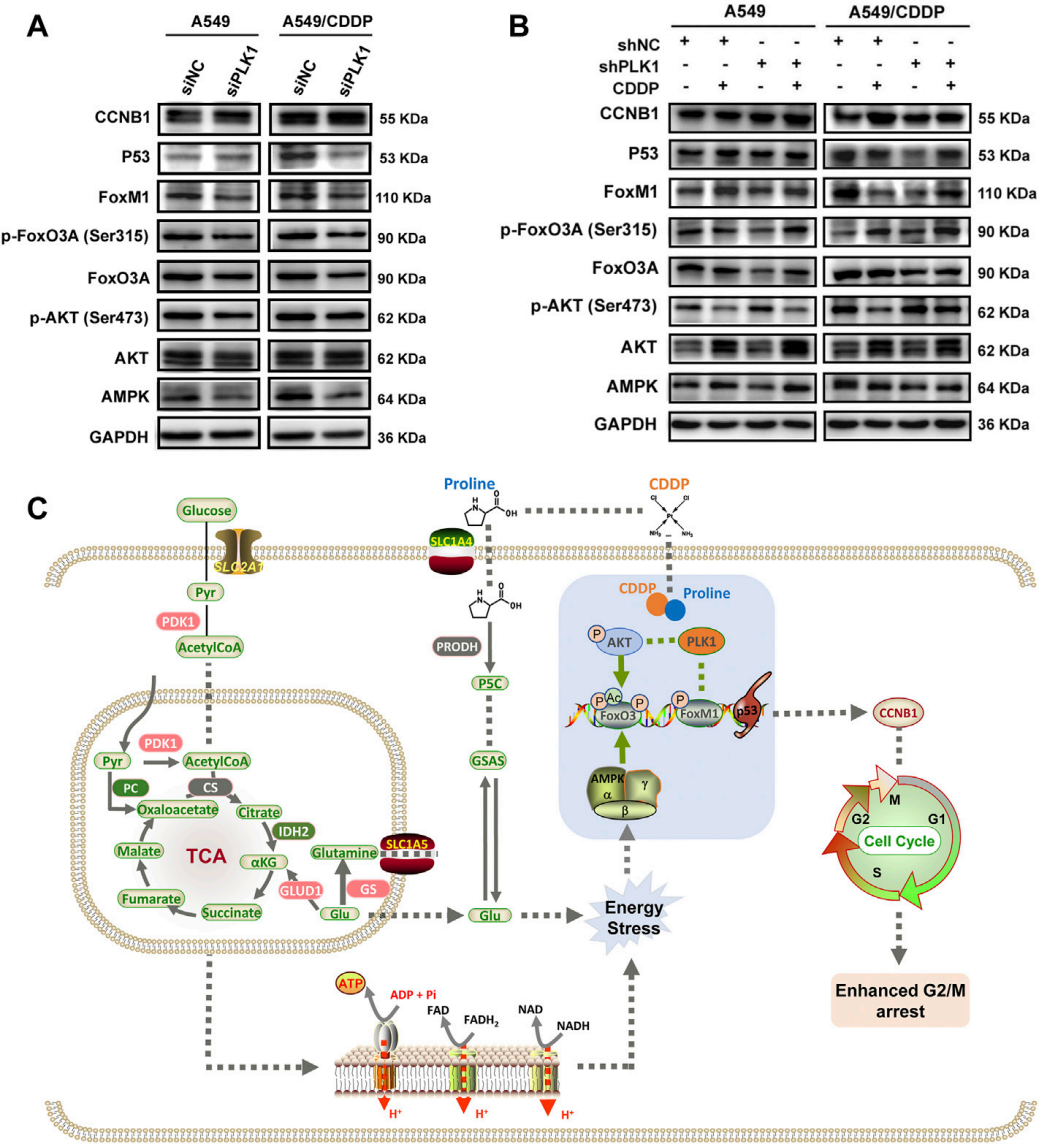
The expression levels of markers involved in cell cycle arrest induced by PLK1 inhibition. **(A)** The levels of AMPK, AKT, p-AKT, FoxO3A, p-FoxO3A, FoxM1, P53, and CCNB1 in the parental and cisplatin-resistant A549 cells infected with PLK1 siRNA. **(B)** The shNC- or shPLK1-transfected A549 and A549/CDDP cells were incubated with 5 μM CDDP to detect the expression levels of PLK1-related proteins. **(C)** Working model showing the underlying mechanism of proline enhanced cisplatin sensitivity in NSCLC cells.

In addition to the cell cycle blockade, the expression levels of upstream and downstream molecules involved in regulating PLK1-mediated cisplatin susceptibility were examined through western blotting analysis. Successful inhibition of PLK1 resulted in a slight decrease in cell proliferation, which was detected by the EdU proliferation assay (Supplementary Figures S10F-H). Given the intracellular imbalance in energy metabolism caused by disruption of the TCA cycle and oxidative phosphorylation, we firstly determined AMPK, a primary regulator of cellular energy homeostasis, showed significantly reduced expression levels in A549 and A549/CDDP cells transfected with PLK1 siRNA (Supplementary Figure S12B, Supplementary Figures S12C). In addition, the PLK1 expression forms positive feedback on phosphorylation of FOXM1 activity, and the transcriptional activity of FOXO3A is modulated by AMPK (Fu et al., 2008; Chiacchiera and Simone, 2010). The FOXO3A-FOXM1 axis plays a pivotal role in DNA damage response and drug resistance (de Moraes et al., 2016). The results showed that si-PLK1 significantly repressed the expression of FOXO3A, p-FOXO3A, FOXM1, and P53, especially in A549/CDDP cells (Figure 6A).

Incubation of PLK1 shRNA-transfected cells with 5 μM CDDP resulted in decreased protein expression of p-AKT and increased AMPK, AKT, p-FOXO3A, and FOXM1, whereas the protein level of FOXO3A had no significant difference. These results might indicate a stressful response to cisplatin after depletion of PLK1 in both A549 and A549/CDDP cells. In contrast, cells transfected with control shRNA and PLK1 shRNA showed decreased phosphorylation levels of AKT and increased phosphorylation levels of FOXO3A under CDDP treatment, implying that cisplatin inhibited P13K/AKT pathway and activated FOXO3A in PLK1-depleted cells. As a downstream signaling molecule of AKT, the protein level of P53 was significantly increased, which ultimately led to cell growth inhibition and cellular apoptosis. Cisplatin upregulated FOXM1, a cell cycle regulator, and FOXM1 cyclically increased CCNB1 to regulate the cell mitotic progression in cells transfected with PLK1 shRNA (Figure 6B). These findings confirmed that cisplatin altered the expression levels of upstream and downstream signaling molecules associated with PLK1, which ultimately affected cell mitotic progression, leading to cell growth inhibition and cellular apoptosis (Figure 6C).

## 4 Discussion

Despite progress in cancer therapeutics, chemotherapy outcomes and prognosis of NSCLC remains poor, primarily due to the development of cisplatin resistance (Olaussen et al., 2006). According to studies, cisplatin resistance is associated with reduced cellular accumulation, increased glutathione clearance, DNA damage repair, and relative signal transduction pathways (Galluzzi et al., 2012). There remains a lack of effective strategies to reverse the resistance in NSCLC cells. Recently, metabolic reprogramming has gained increasing attention in chemoresistance. Accumulating evidence suggests that changes in metabolic processes, like glycolysis and pentose phosphate pathway, glutamine metabolism, amino acids metabolism and lipid metabolic pathway, greatly contribute to the cisplatin sensitivity of cancer cells (Cocetta et al., 2020). Among these, amino acid metabolism serves as a promising target for cancer therapy, whereas the role of amino acids in cisplatin resistance is still under exploration. In our study, the level of most amino acids significantly decreased in cisplatin-resistant A549 cells compared with the parental cells as detected by targeted metabolomics, implying a high rate of protein synthesis (Shi et al., 2019).

Glutathione metabolism is an essential pathway in regulating cisplatin resistance, associated with increased cellular glutathione and glutamate (Godwin et al., 1992; Guo et al., 2021). The glutamate was significantly elevated in A549/CDDP cells in our study, whereas the glutamine concentration had no difference. We hypothesized that the cisplatin-resistant cells absorb glutamine in the culture medium and convert it to glutamate to meet their high energy requirements (Butler et al., 2021). Since our studies showed that altered metabolic response led to the modulation of cellular status and drug efficiency (Wang et al., 2019), the supplementation of exogenous proline markedly enhanced the cellular sensitivity to cisplatin in both NSCLC cells and ovarian cancer cells. It has been demonstrated that proline was selected as an alternative energy source to reprogram the cellular metabolism when a limited glucose supply is available (Huynh et al., 2020; Patriarca et al., 2021). In addition, reducing proline may limit the growth of tumours with unresolved ER stress (Sahu et al., 2016). However, the role of proline in cancer resistance remains unclear. Phenotypically, combined treatment with proline and CDDP inhibited cell growth and proliferation, cell cycle arrest and increased cell apoptosis, indicating that exogenous metabolic stimuli could modulate the cisplatin resistance of cancer cells.

The transcriptome provides more information about molecular changes involved in cisplatin susceptibility. Indeed, we identified the most enriched pathways under the treatment of proline and cisplatin by transcriptomic analysis, including the upregulated FoxO signaling pathway and the down-regulated cell cycle pathway. In previous studies, PLK1 expression was associated with drug resistance in various cancer cells, and a high expression level of PLK1 was associated with a poor prognosis in NSCLC through the Kaplan-Meier plotter analysis (Supplementary Figure S13). Inhibition of PLK1 overcomes therapeutic resistance to BET inhibitors in triple-negative breast cancer (Zhu et al., 2020). Furthermore, inhibition of PLK1 by inhibitor BI2536 enhanced the CDDP sensitivity of oesophageal squamous cell carcinoma and gastric cancer cells (Chen et al., 2019; Wu et al., 2019). Our results suggested that the exogenous addition of proline had no direct correlation with changes in PLK1 expression. The results of transcriptome profiles indicated that the transcription level of PLK1 in A549 and A549/CDDP cells treated with proline alone did not change significantly compared to the control group. We observed downregulated gene and protein expression levels of PLK1 only when cells were treated with proline and cisplatin simultaneously. In addition, depletion of PLK1 induced cell cycle arrest *in vitro* and exhibited efficient anticancer effects against NSCLC *in vivo*.

In addition to cell cycle, the expression levels of genes involved in proline metabolism, such as *PYCR1, PYCR2, PYCR3, ALDH4A1, GLUD1, ALDH18A1,* and *GLS*, were significantly increased in A549/CDDP cells exposed to the combination of proline and CDDP (Supplementary Figure S12A). In previous studies, proline metabolism plays a key role in cancer development and progression, and the overexpression of PYCRs and ALDH18A1 has been associated with a poor clinical course (D'Aniello et al., 2020). In the current work, the mRNA levels of *PYCR2* and *ALDH18A1* decreased in A549/CDDP cells compared to A549 cells, while increased in cells treated with proline or the combination, implying PYCR2 and ALDH18A1 might contribute to enhancing cisplatin sensitivity. Meanwhile, the expression level of cytosolic PYCR3 increased only in cisplatin-resistant cells treated with proline and further upregulated under the combined action of proline and CDDP, suggesting enhanced proline synthesis. The degradation of proline was catalyzed by proline dehydrogenase/proline oxidase (PRODH/POX), whereas the PRODH expression had no significant difference from the CDDP treatment group, suggesting that proline oxidation to P5C in mitochondria may not be affected. The above results illustrated the accumulation of proline, especially in A549/CDDP cells. Cells used proline to produce ATP and ROS, while proline itself has been shown to enhance cellular ROS scavenging (Krishnan et al., 2008; Tanner et al., 2018). Thus, we observed a slight increase in ROS production of A549/CDDP cells treated with proline and CDDP, which might also account for the enhanced susceptibility to cisplatin (Supplementary Figure S14). Moreover, P5C in proline metabolism served as a fuel for the TCA cycle, and the expression profiles of most genes involved in the TCA cycle and OXPHOS were significantly changed, implying an imbalance in ATP level (Supplementary Figure S12B, Supplementary Figure S12C). Among them, the expression level of *SUCLG2* was upregulated in A549/CDDP cells compared to the parental cells, while significantly decreased after treatment with proline and CDDP. *SUCLG2* deficiency resulted in the reversal of TCA cycle and the accumulation of citrate and AcCoA, interfering with ATP production (Wu et al., 2020). The decreased cellular ATP content under the combination treatment of proline and CDDP confirmed the conclusion, thus ATP content was insufficient to sustain cell growth and proliferation.

We then examined underlying molecular mechanisms by which PLK1 regulated the cisplatin susceptibility in NSCLC. We discovered that the knockdown of PLK1 inhibited the expression of AMPK a key regulator for cellular energy homeostasis, whereas the addition of CDDP activated AMPK (Xiao et al., 2013; Lee et al., 2015). The activation of the energy sensor pathway by metabolic stimuli leads to AMPK-mediated phosphorylation of FOXO3A to regulate glucose metabolism and oxidative stress, and AMPK induced the phosphorylation of P53 and initiated an AMPK-dependent cell cycle arrest (Jones et al., 2005; Greer et al., 2007; An et al., 2020). Consistent with these studies, the expression of FOXO3A, p-FOXO3A and P53 in cells exposed to CDDP increased to maintain the cellular energy balance. In addition to the P53 signaling pathway, the FOXO3A-FOXM1 axis consists of two forkhead transcription factors that play a critical role in DNA damage response, cell cycle regulation and genotoxic drug resistance (de Moraes et al., 2016). FOXM1 acted as a substrate of PLK1, regulating PLK1-dependent cell cycle progression through the transcriptional program (Fu et al., 2008). In addition, CCNB1 was another target gene of FOXM1 (Dibb et al., 2012). The results confirmed that FOXM1 activity was reduced by depletion of PLK1 and increased upon cisplatin treatment, accompanied by the increase of CCNB1, ultimately resulting in cell cycle arrest in NSCLC cells with PLK1 deficit. Furthermore, the expression levels of AKT increased in the presence of CDDP accompanied by a decrease of phosphorylated AKT level, indicating that the PI3K/AKT signaling pathway was inhibited upon cisplatin treatment, which might be related to CDDP-induced apoptosis and remained to be further explored (Liao et al., 2020). All above results suggested that PLK1 reduced cisplatin sensitivity in NSCLC cells by regulating multiple signaling molecules.

In conclusion, our results showed that the cisplatin susceptibility of NSCLC cells could be modulated by metabolic reprogramming. Proline in amino acid metabolism exerted efficiently inhibitory effects on both the parental and cisplatin-resistant cells when combined with CDDP, as confirmed *in vitro* and xenograft models of nude mice. According to the transcriptome analysis, the FoxO signaling pathway and cell cycle pathway were significantly enriched in A549/CDDP cells exposed to exogenous proline supplementation. In addition to the disturbed energy metabolism, cell cycle arrest at G2/M induced by a significant reduction of PLK1 was considered a feature to overcome cisplatin resistance. Thus, our findings provide novel insights into the role of PLK1 in cisplatin resistance of NSCLC and offer new co-therapeutic strategies to address cisplatin resistance in clinical treatment.

## Data Availability

The datasets presented in this study can be found in online repositories. The names of the repository/repositories and accession number(s) can be found below: https://www.ncbi.nlm.nih.gov; PRJNA838591.
