# Supplementary material for: Exogenous proline enhances susceptibility of NSCLC to cisplatin via metabolic reprogramming and PLK1-mediated cell cycle arrest
Source: Front Pharmacol. 2022 Jul 14;13:942261. doi: 10.3389/fphar.2022.942261 (PMC9330219; doi:10.3389/fphar.2022.942261)
Supplement: Supplementary file 1 [file DataSheet1.docx]

Supplementary Material

# Supplementary Materials and Methods

- 1. **Drugs**

Cisplatin and the tetrazolium dye 3-(4,5-dimethylthiazol-2-yl)-2,5-diphenyltetrazolium bromide (MTT) (M2003, CAS Number: 57360-69-7) were purchased from Sigma (Germany). HPLC grade methanol and acetonitrile were purchased from Merck Company (Germany). BCA assay kit was provided by Beyotime Biotechnology Corporation (Shanghai, China). BI2536 was purchased from Selleckchem.

- 1. **Cell viability assay**

MTT assay was performed for evaluating cell viability. All kinds of cells were seeded in 96-well plates at 3000 cells per well. After 24 hrs, different concentrations of cisplatin (0-200 μM) and 20 mM metabolites were added to the plate and then incubated for another 72 hrs at 37 °C. After that, cells were treated with the MTT for 4 hrs. Finally, upon resolving the crystal with 100 μL of DMSO, the absorbance at 570 nm was measured by a multifunctional microplate reader (SpectraMAX i3x, MD). The IC50 values were calculated from the average viability curves generated by three independent assays.

- 1. **EdU cell proliferation assay**

For the EdU assay, the experiment was conducted according to the protocol of Cell-Light EdU Apollo567 In Vitro Kit (RIBOBIO, Guangzhou, China). A549 and A549/CDDP cells were seeded in 12-well plates with a density of 5 × 104 cells per well. After incubation for 24 hrs, 10 μM CDDP, 20 mM proline or their combination were added to the plate and incubated for another 48 hrs. Then RPMI1640 medium with 50 μM EdU was added into samples and incubated for 2 hrs, followed by washing twice in PBS and DAPI staining. All groups of samples were then detected by laser scanning confocal microscopy (LSM 980, ZEISS).

- 1. **Flow cytometry analysis of cell apoptosis**

A549 and A549/CDDP cells were seeded in 6-well plates at a density of 15 × 104 cells per well. 5 μM CDDP, 20 mM proline and the combination were added to the plate. After incubation for 48 hrs, cells were harvested and stained with Annexin V-FITC and propidium iodide (PI) for 15 min in a dark place through an apoptosis detection kit (BD Pharmingen). Finally, the stained cells were analyzed with the flow cytometer (FACSCelestaTM, BD).

- 1. **Flow cytometry analysis of cell cycle**

The procedure of cell pretreatment was the same as that of cell apoptosis detection. Cells were harvested and washed three times with PBS, and then immobilized for 24 hrs using 70% ethanol. After that, cell staining was performed according to the manufacturer’s instructions (FxCycleTM PI/RNase Staining Solution, Thermo Fisher, #F10797). In brief, 0.5 mL staining solution was added to each flow cytometry sample and incubated for 15-30 min at RT, then the samples were analyzed by flow cytometer (FACSCelestaTM, BD).

- 1. **Measurement of ATP**

Cellular ATP concentration of cells exposed to CDDP, proline, and the combination treatment were measured with BacTiter-Glo Microbial Cell Viability Assay Kit (Promega) according to the manufacturer’s instruction. In brief, 100 μL of A549 and A549/CDDP cells were seeded in 96-well plates with white background at 2000 cells per well. After being treated with drugs for 72 hrs, the same volume of CellTiter-Glo reagent was added to the wells. The mixture was incubated for 15 min at room temperature, and then the luminescence was detected by a multifunctional microplate reader (SpectraMAX i3x, MD). Three biological repeats were performed in each group.

- 1. **Quantitative real-time PCR**

The total RNA of cells in 6-well plates was extracted using TRIzol reagent (Invitrogen). The RNA concentration and purity were estimated with NanoDrop 2000 (Thermo Scientific, Waltham, MA) by measuring absorbance at 260 nm and 280 nm, respectively. The total RNA was used to synthesize cDNAs using HiScript III RT SuperMix for qPCR (Vazyme) according to the manufacturer’s instruction. Then, the samples of qRT-PCR were prepared using ChamQ SYBR qPCR Master Mix (Vazyme) in 20 μL reaction volume and detected by QuantStudio 3 (Applied Biosystems, Foster City, CA). GAPDH was used as an internal reference and the relative expression of target mRNA (Table S1) was calculated by 2-△△Ct in three replications. All primer sequences are listed in Supplementary Table S1.

- 1. **Western blotting**

The cells in 6-well plates were first lysed with RIPA buffer containing 1 mM PMSF for 30 min on ice and then centrifuged at 12,000 g for 15 min at 4 °C. The supernatant was transferred and the concentration was determined by BCA assay (Beyotime). Protein was separated by SDS-PAGE gels and the transferred membrane was blocked in 5% skimmed milk for 1 h and then incubated with primary antibodies at 4 °C overnight. After being washed with TBST for 7 min thrice, the membranes were incubated with secondary antibodies for 1 h at room temperature. After washing, the protein expression was visualized via Amersham Imager 680 (GE). The protein expression level was analyzed using the ImageJ software. The primary antibody for PLK1 (#ab70697, RRID: AB_1269816), p-PLK1 (T210) (#ab39068, RRID: AB_10861033), P53 (#ab32389, RRID: AB_776981) were purchased from Abcam. AMPK (#66536, RRID:AB_2881899), AKT (#60203, RRID: AB_10919160), p-AKT (#66444, RRID: AB_2782958), FoxO3A (#66428, RRID: AB_2881799), p-FoxO3A (#28755, RRID: AB_2881210), FoxM1 (#13147, RRID: AB_2106213), CCNB1 (#28603, RRID: AB_2881179) were purchased from Proteintech Group.

# Supplementary Figures and Tables

## Supplementary Figures


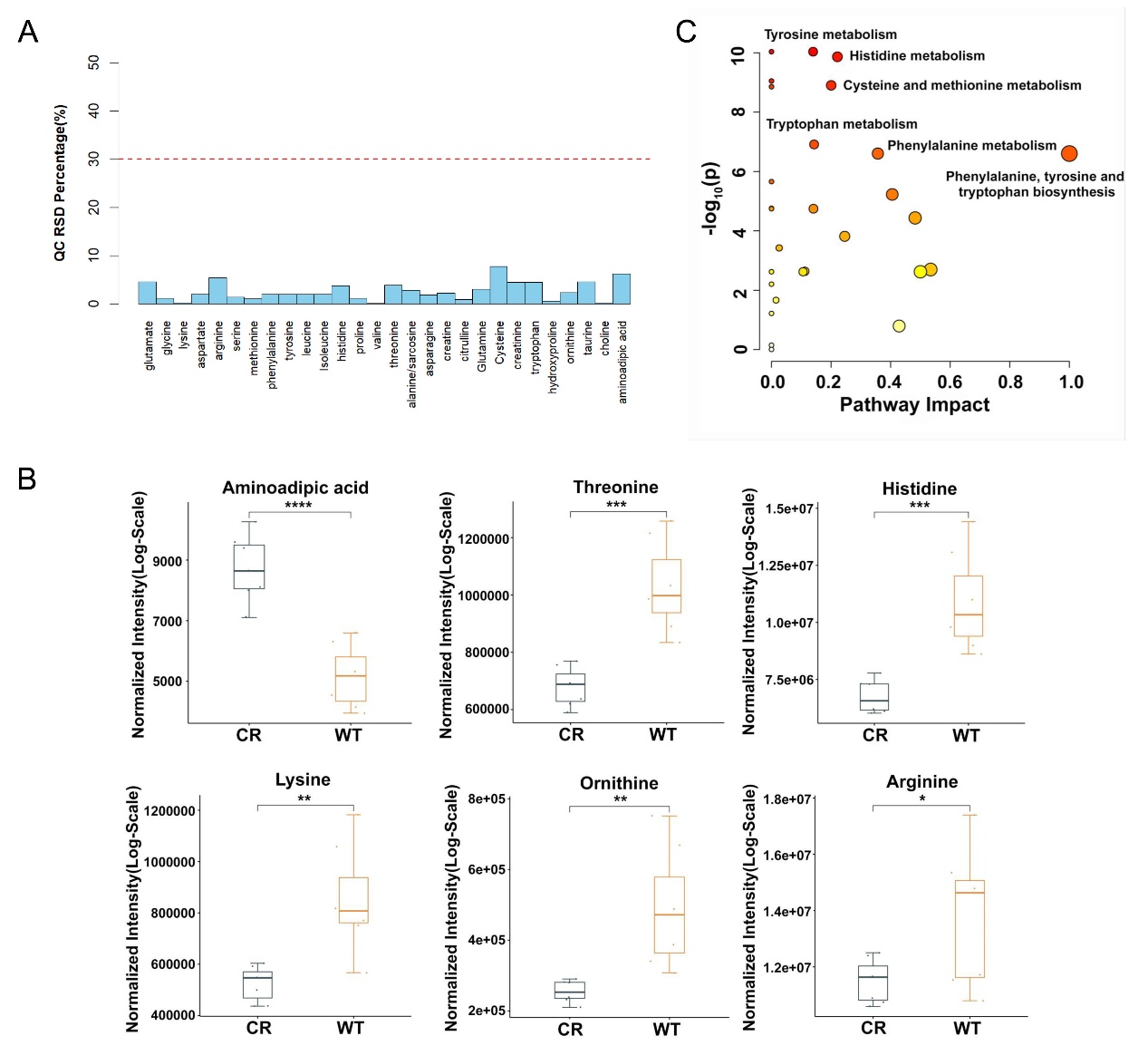


**Supplementary Figure 1.** The analysis of targeted amino acids metabolomics. (A) The RSD distribution of QC samples for quality control. (B) The relative abundance of other significantly differential amino acids. (C) The KEGG pathways enriched by 28 kinds of detected amino acids.


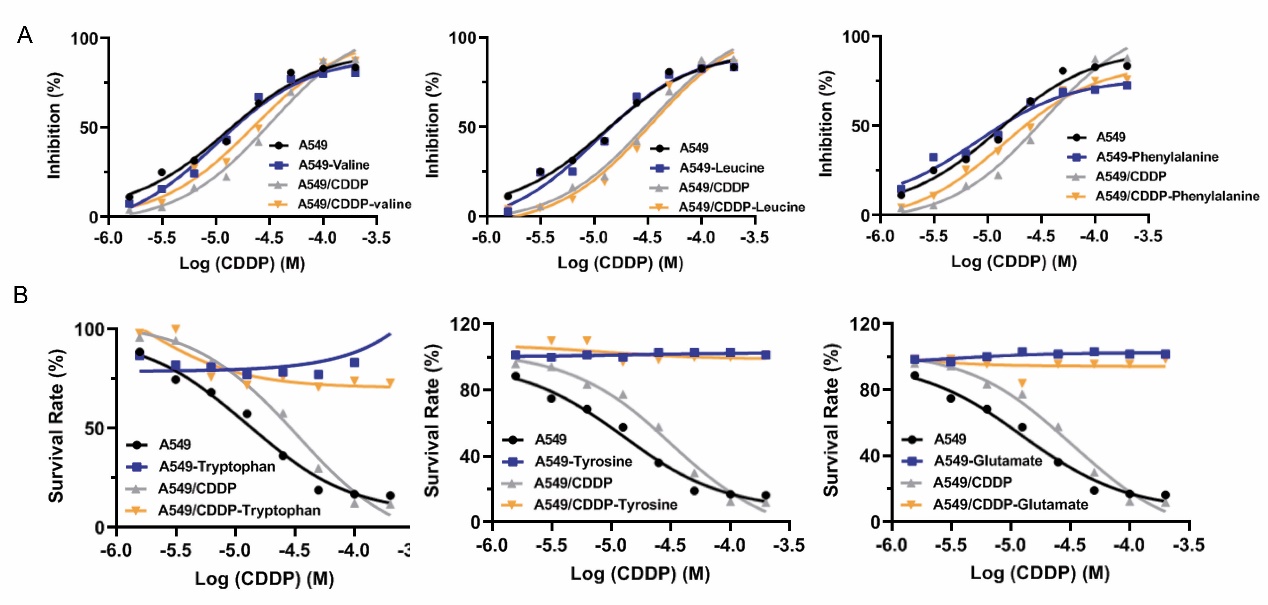


**Supplementary Figure 2.** Effect of exogenous amino acids on the susceptibility of A549 and A549/CDDP cells to CDDP treatment. (A) Valine, leucine and phenylalanine. (B) Tryptophan, tyrosine and glutamate.


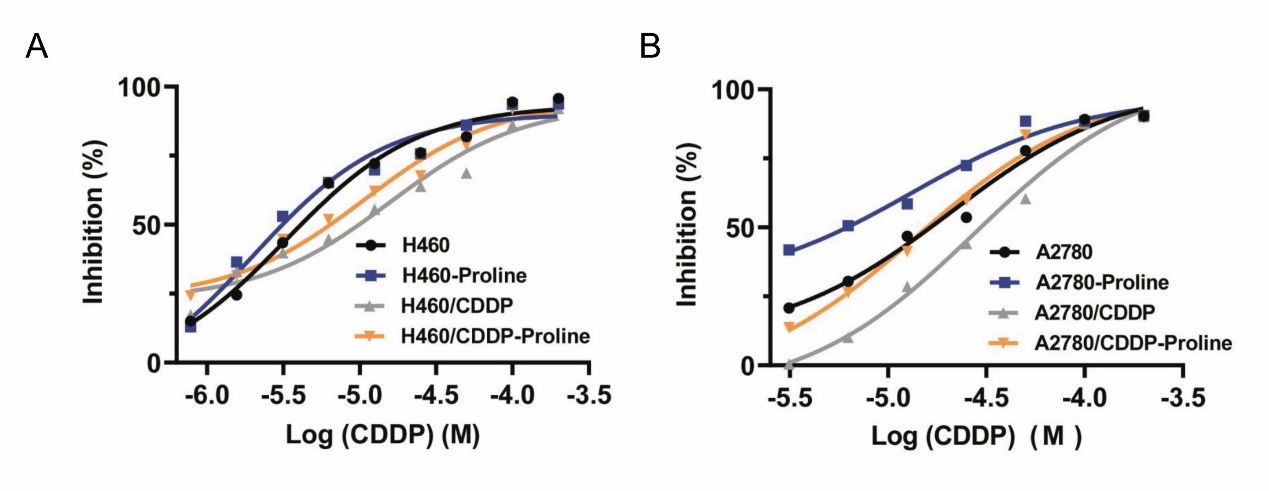


**Supplementary Figure 3.** The effects of exogenous 20 mM proline combined with CDDP on different cell lines. (A) H460 and H460/CDDP cells. (B) A2780 and A2780/CDDP cells.


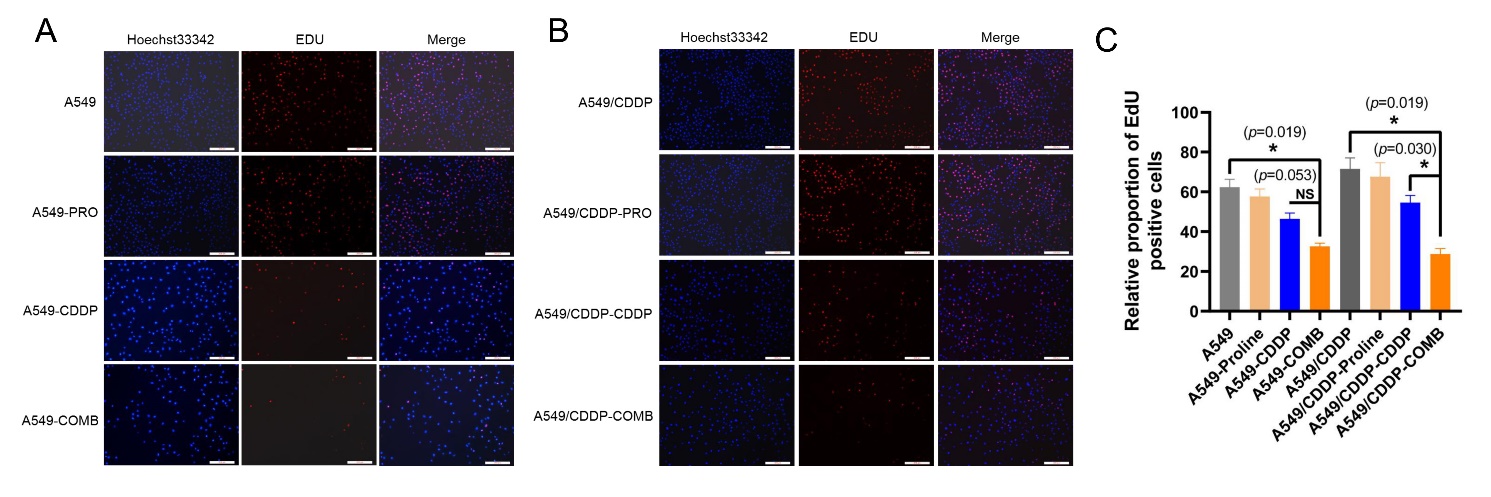


**Supplementary Figure 4.** EdU assay for detecting cell proliferation. (A) DNA replication activity of A549 cells after treatment with proline (20 mM), CDDP (10 μM) or their combinations for 48 hrs. (B) A549/CDDP cells were treated in the same way as A549 cells. (C) The quantification of the ratio of EdU-positive cells to the total number of Hoechst-positive cells in (A) and (B). Data are expressed as mean ± SEM, *P < 0.05, NS: No Significance.


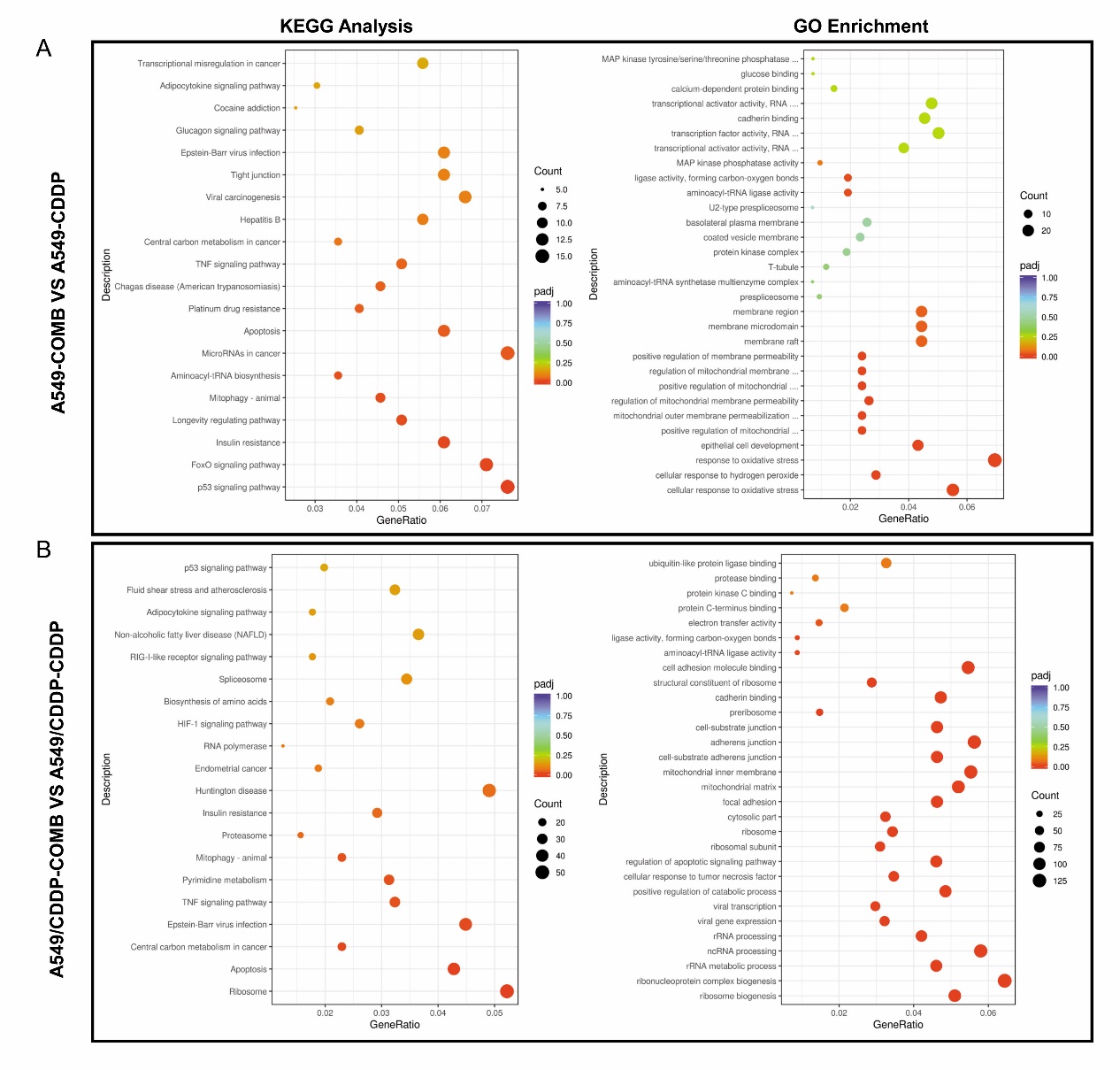


**Supplementary Figure 5.** GO enrichment and KEGG pathway analysis of significantly differential up-regulation genes between different treatment groups of A549 and A549/CDDP cells. (A) Pathways defined by up-regulation genes between the CDDP treatment group and the combination treatment group (proline and CDDP) of A549 cell lines. (B) Pathway analysis of increased genes identified in combination group versus the CDDP treated group of A549/CDDP cells.


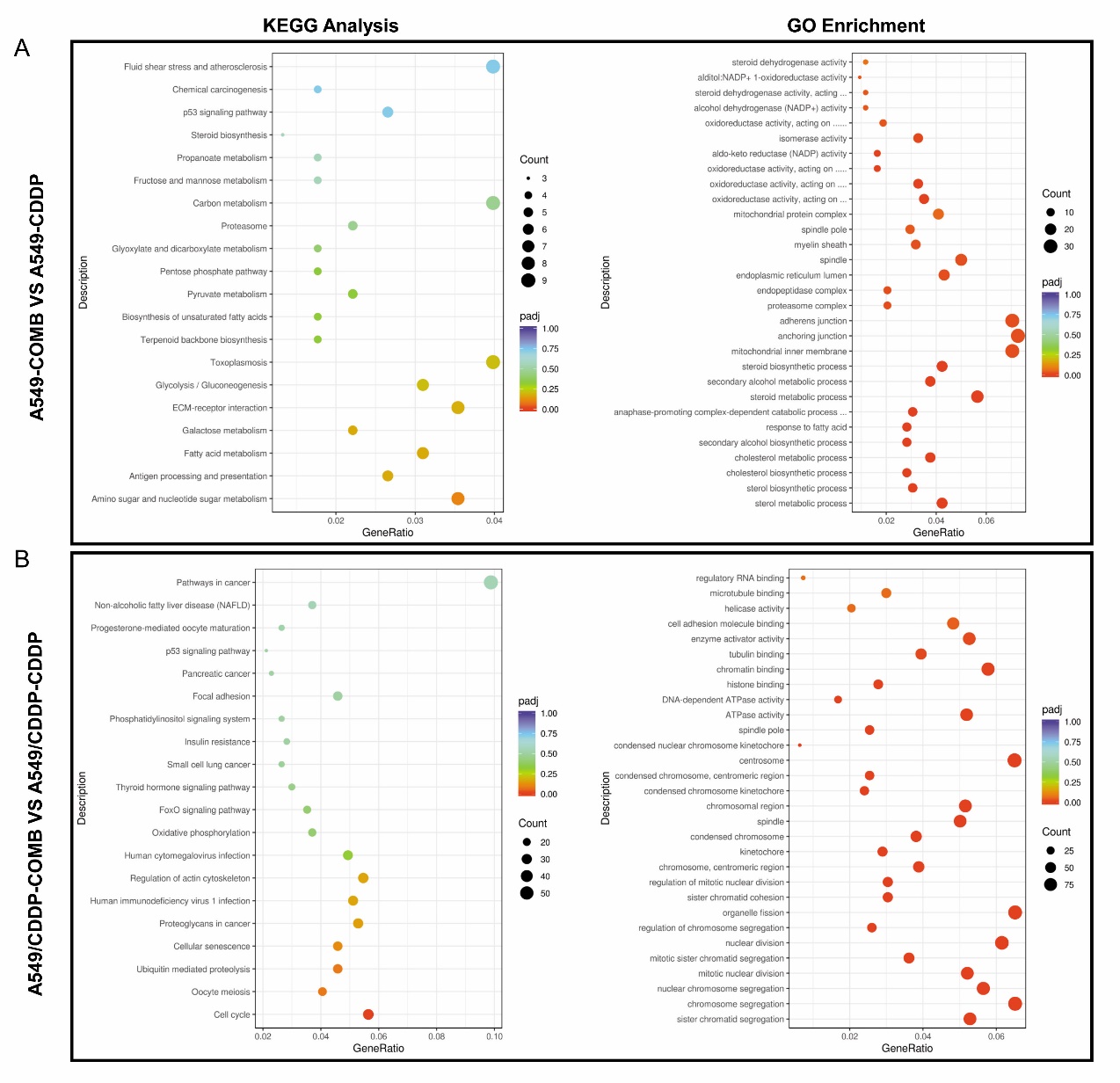


**Supplementary Figure 6.** GO enrichment and KEGG pathway analysis of significantly differential down-regulation genes between different treatment groups of A549 and A549/CDDP cells. (A) Pathways enrichment by down-regulation genes obtained from the combination treatment group versus the CDDP treatment group of A549 cell lines. (B) Pathway analysis of decreased genes identified in combination group versus the CDDP treated group of A549/CDDP cells.


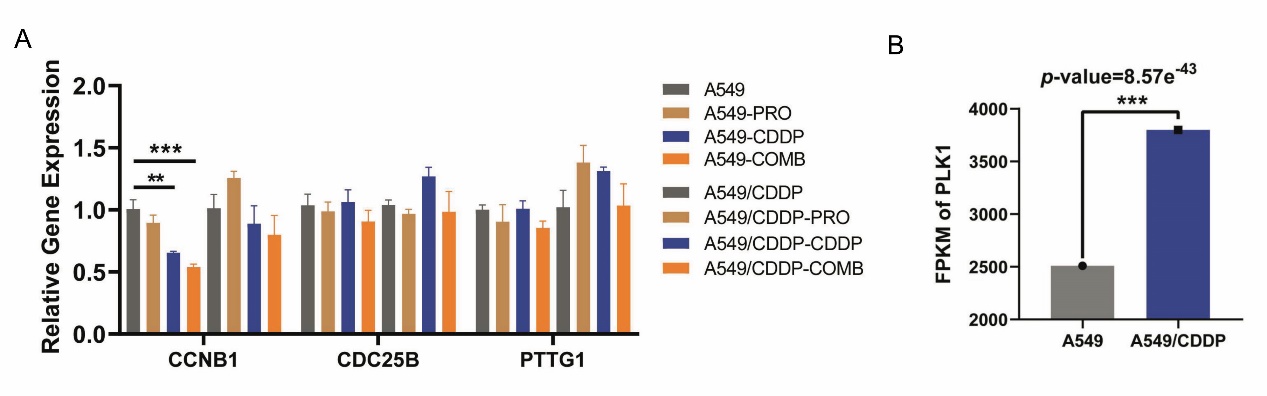


**Supplementary Figure 7.** Relative transcriptions of cell cycle genes in A549 and A549/CDDP cells treated with 20 mM proline, 10 μM CDDP or their combination for 48 hrs. (A) The changes of significantly differential genes involved in the cell cycle pathway were verified by qRT-PCR. (B) Relative abundance of PLK1 derived from transcriptomics.


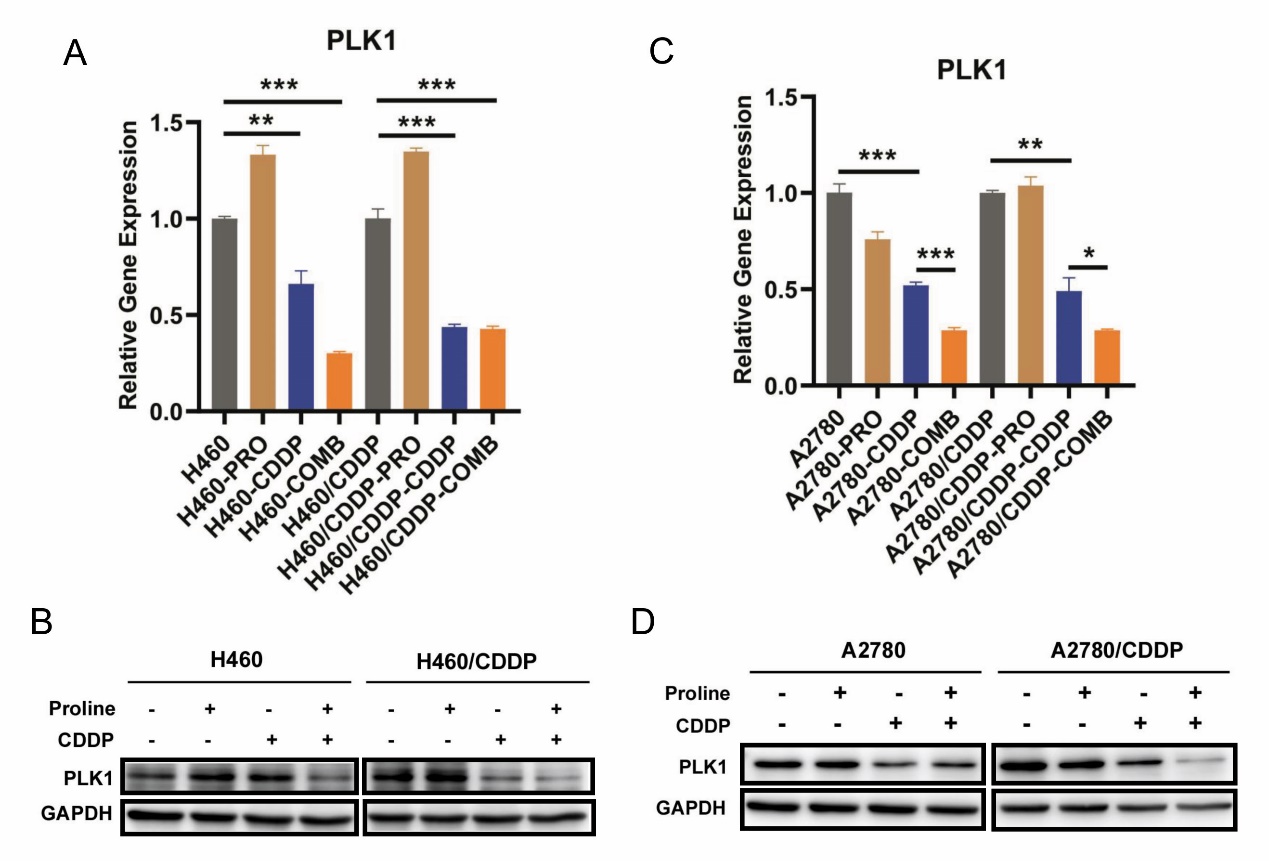


**Supplementary Figure 8.** Gene and protein expression of PLK1 detected by qRT-PCR and western blot, respectively. (A) Relative gene expression of PLK1 in H460 and H460/CDPP cells treated with proline (20 mM), CDDP (5 μM) or their combination. (B) Western blotting to compare PLK1 expression in H460 and H460/CDDP cell lines under different treatments. (C) The same detection of PLK1 as (A) in A2780 and A2780/CDDP cells. (D) The similar determination of PLK1 protein expression to (B) in A2780 and A2780/CDDP cells.


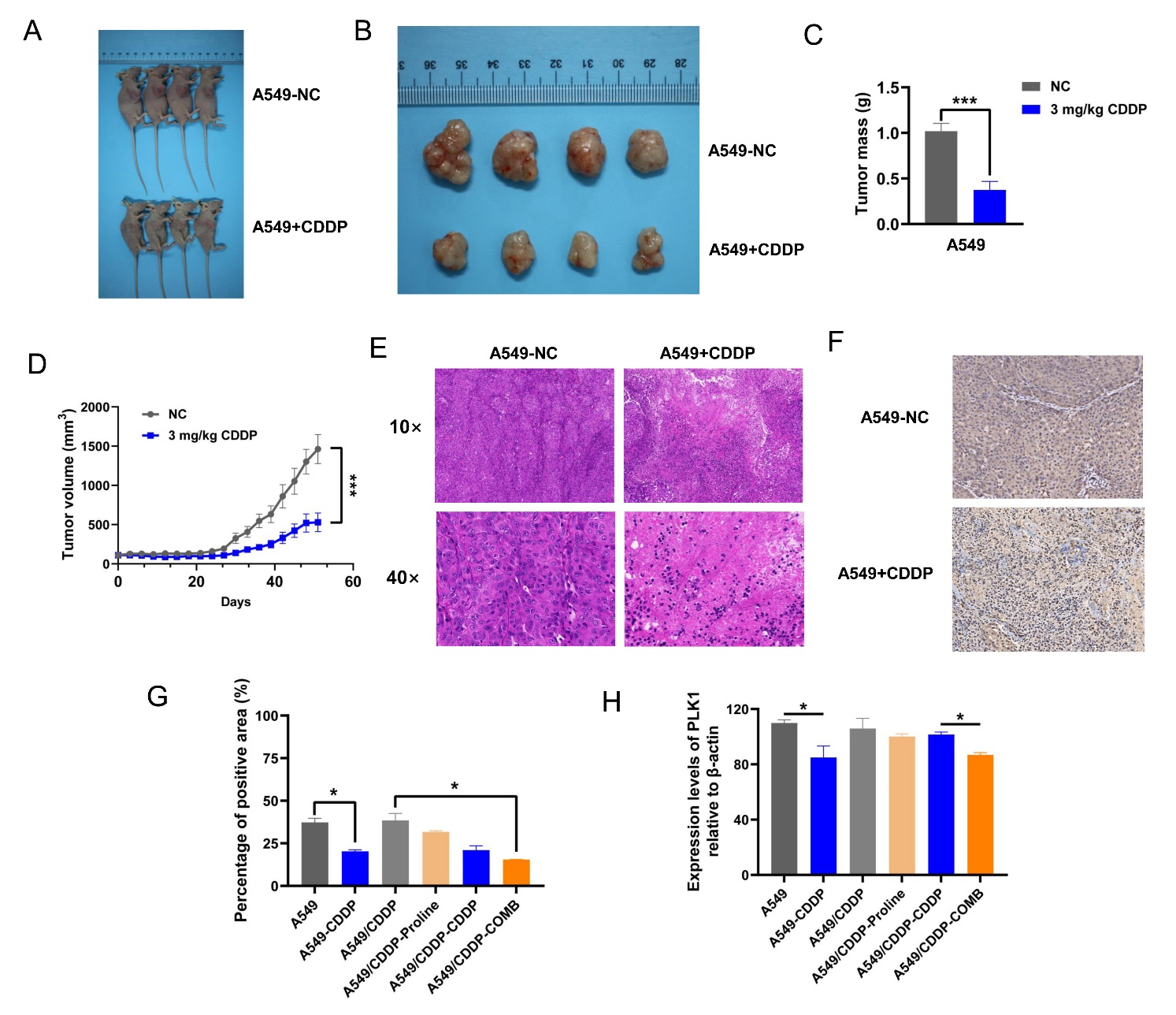


**Supplementary Figure 9.** Cisplatin inhibited the growth of the xenograft tumor model of A549 cells. (A) Representative images of tumor-bearing mice. The mice were collected 7 weeks after implantation (n=4). (B) Tumors from mice. (C) Tumor mass. (D) The growth kinetics of tumor volume of nude mice from the control group and 3 mg/kg cisplatin-treatment group were measured and calculated as volume = (width^2^ × length)/2. (E) Representative results of HE staining analysis in tumor tissues. (F) Tissue sections of tumors were conducted PLK1 analysis by IHC staining. (G) Quantification of PLK1 signal in representative IHC stained sections of tumor tissues. (H) Quantification of protein expression of PLK1 relative to β-actin in xenografts. Data are expressed as mean ± SEM, *P < 0.05, **P <0.01, ***P <0.001.


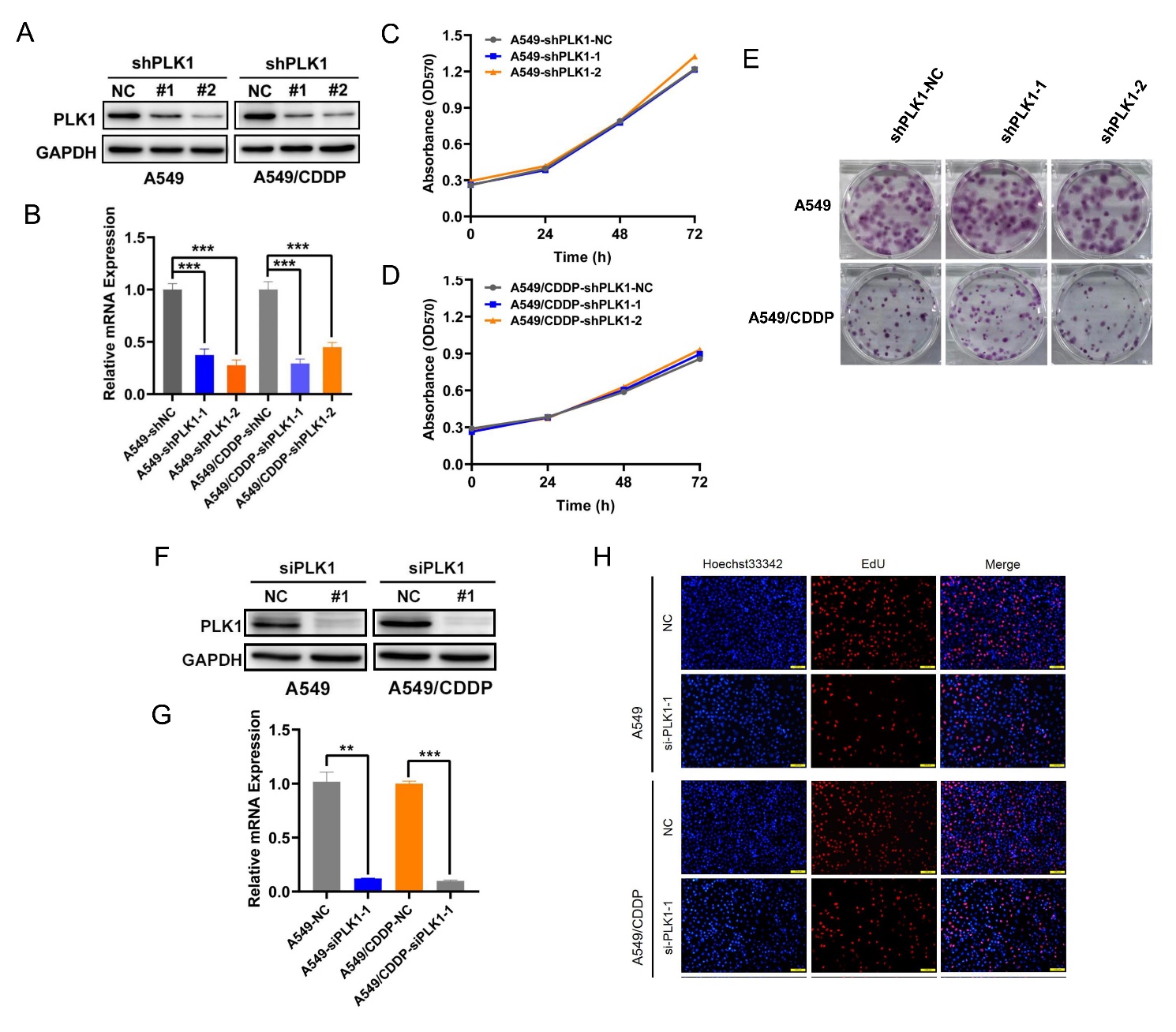


**Supplementary Figure 10.** Effects of PLK1 shRNA and siRNA. (A) Western blot analysis for PLK1 expression in A549 and A549/CDDP cells transfected with pLKO5 empty vector and PLK1 shRNA. (B) Verification of PLK1 mRNA expression in A549 and A549/CDDP cells using qRT-PCR. (C) Growth curves of A549 cells stably transfected with pLKO5 empty vector and PLK1 shRNA. (D) Determination of growth curves for A549/CDDP cells. (E) Cell cloning capability of stabilized knock-down A549 and A549/CDDP cells detected by single-cell clone assay. (F) Protein expression of PLK1 in A549 and A549/CDDP cells transiently transfected with scrambled control or PLK1 siRNA. (G) Relative transcriptions of siPLK1 were detected by qRT-PCR. (H) EdU assay detected the cell proliferation of A549 and A549/CDDP cells transiently transfected with scrambled control or PLK1 siRNA.


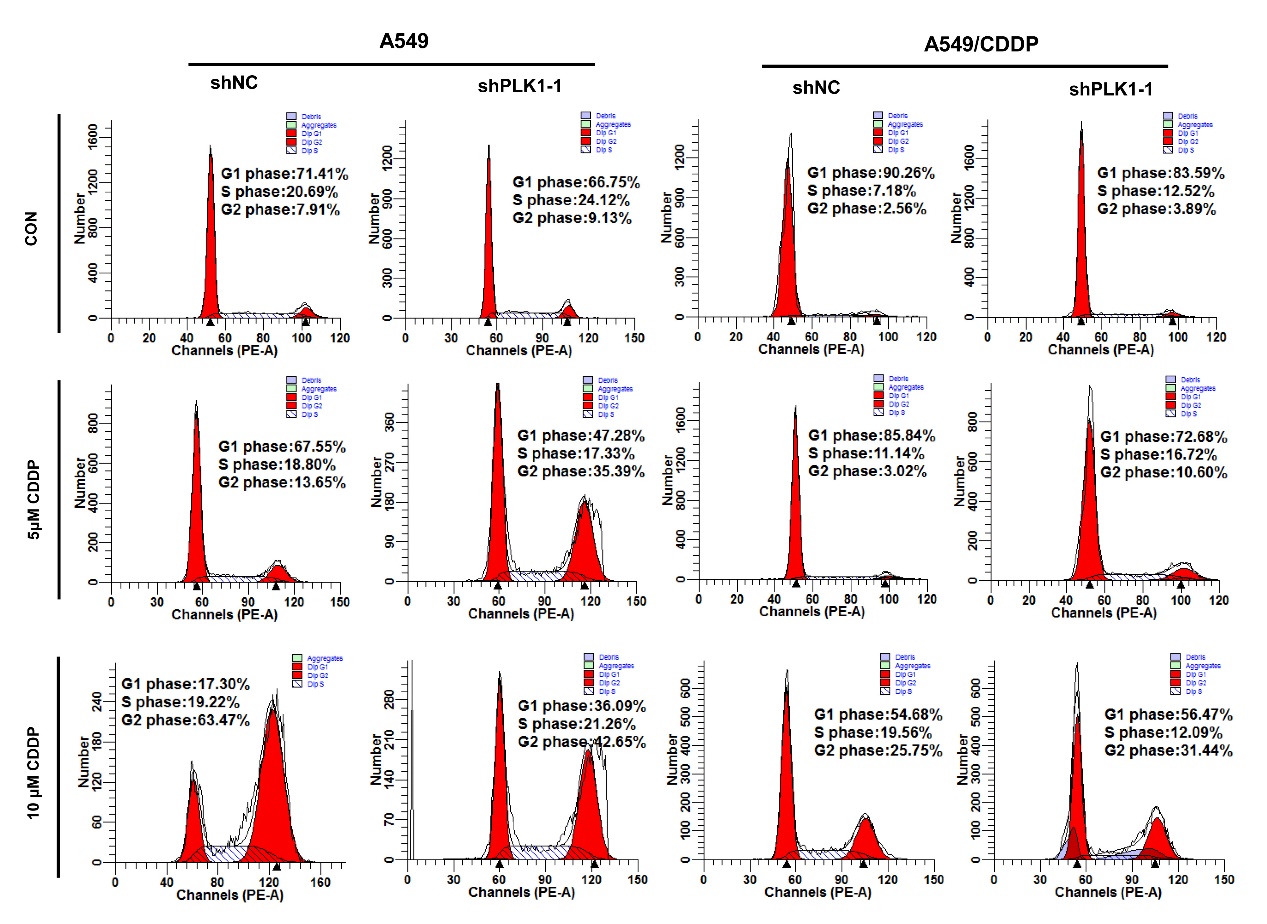


**Supplementary Figure 11.** Cell cycle distribution in A549 and A549/CDDP cells transfected with pLKO5 empty vector and PLK1 shRNA under 5 μM or 10 μM cisplatin treatment.


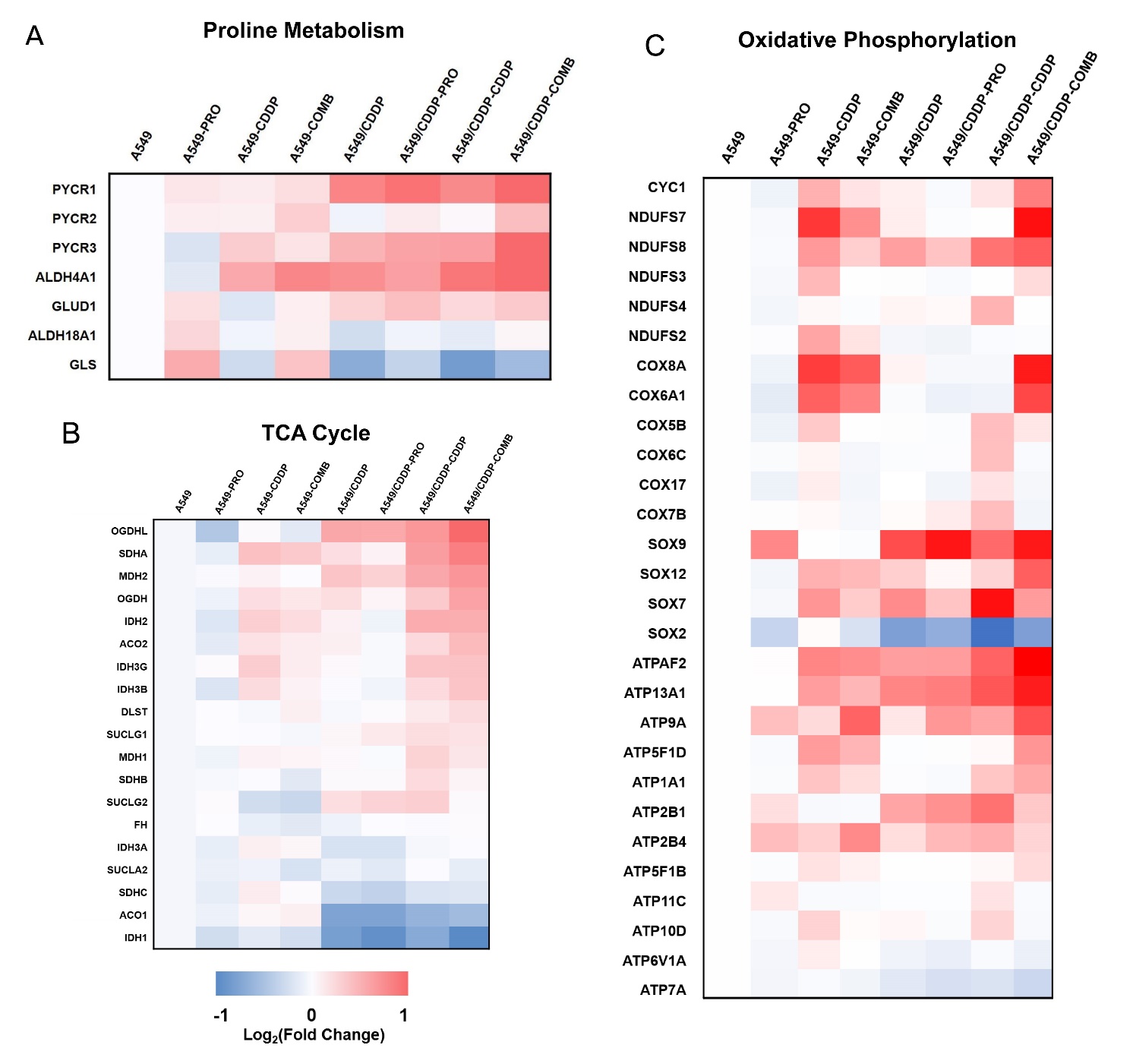


**Supplementary Figure 12.** Gene transcription profiles from A549 and A549/CDDP cells treated with 20 mM proline, 10 μM CDDP or their combination for 24 hrs. Values of three biological replications were reported as log_2_ (fold change) over untreated control of A549 cells in the mRNA level of genes involved in proline metabolism (A), TCA cycle (B), and oxidative phosphorylation (C).


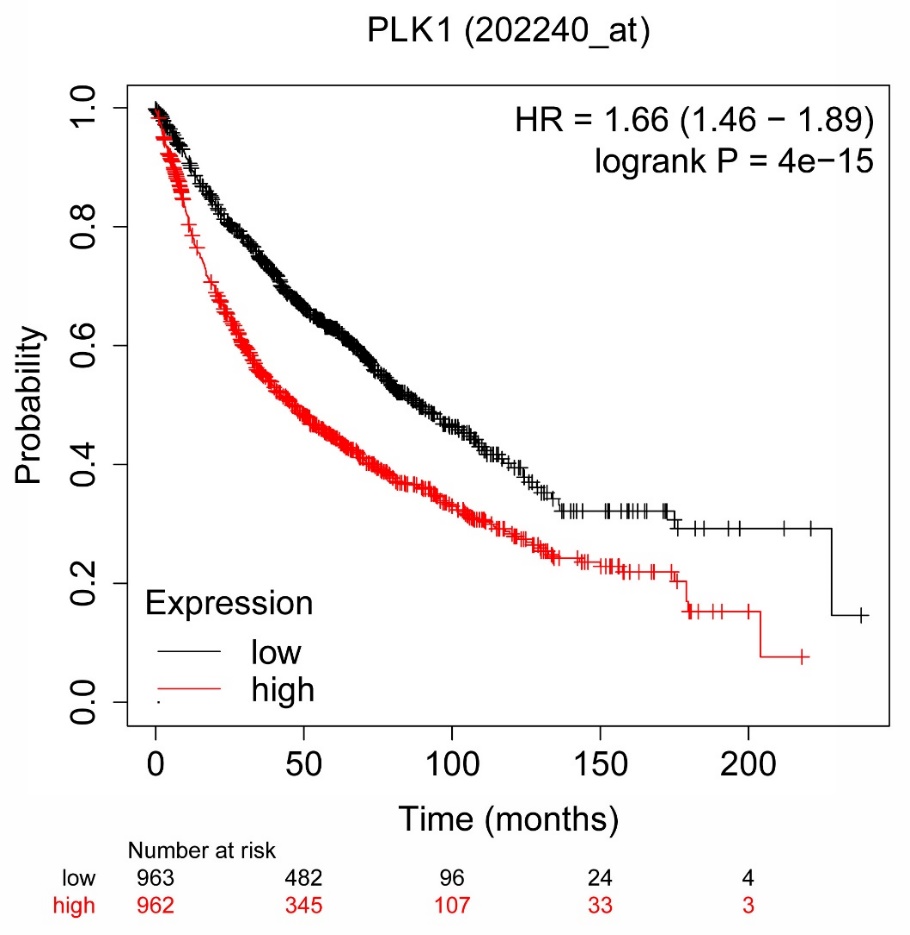


**Supplementary Figure 13.** Linking between PLK1 expression and overall survival (OS) in lung cancer (adenocarcinoma and squamous cell carcinoma) patients (n=1925). The Kaplan-Meier plotter analysis was performed according to the transcriptomic data of NSCLC.


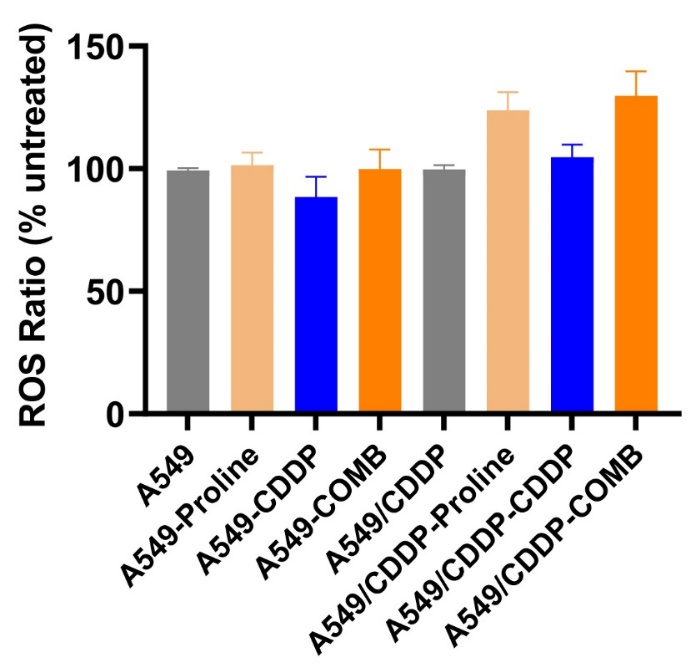


**Supplementary Figure 14.** Relative cellular ROS levels in cisplatin-sensitive and cisplatin-resistant A549 cells under treatment of 20 mM proline, 5 μM CDDP or their combination for 72 hrs.

## Supplementary Tables

**Table. S1** The nucleotide sequences of primers used for qRT-PCR.

| Gene Name | Forward primer (5’-3’) | Reverse primer (5’-3’) |
| --- | --- | --- |
| PLK1  CCNB1  CDC25B  PTTG1  GAPDH | CGAGGACAACGACTTCGTGTT  AAGAGCTTTAAACTTTGGTCTGGG  CACGCCCGTGCAGAATAAGC  TACCTTTGCTTCTCCCACCTTC  CGCTCTCTGCTCCTCCTGTTC | ACAATTTGCCGTAGGTAGTATCG  CTTTGTAAGTCCTTGATTTACCATG  ATGACTCTCTTGTCCAGGCTACAGG  CTAAGGCTTTGATTGAAGGTCCA  ATCCGTTGACTCCGACCTTCAC |

**Table. S2** The target sequences of PLK1.

|  | Sequence |
| --- | --- |
| siPLK1-1  shPLK1-1  shPLK1-2 | GCTCTTCAATGACTCAACA  GTTCTTTACTTCTGGCTATAT  GCTCATCTTGTGCCCACTGAT |
